# Prenatal exposure to essential and toxic elements in relation to infant growth trajectories

**DOI:** 10.1186/s12940-025-01252-w

**Published:** 2026-01-09

**Authors:** Gyeyoon Yim, Brianna C Heggeseth, Diane Gilbert-Diamond, Janet L Peacock, Katerina Margetaki, Emily R Baker, Thomas J Palys, Brian P Jackson, Juliette C Madan, Megan E Romano, Margaret R Karagas, Caitlin G Howe

**Affiliations:** 1 Department of Epidemiology, Geisel School of Medicine, Dartmouth College, Lebanon, NH USA; 2 Department of Mathematics, Statistics, and Computer Science, Macalester College, St Paul, MN USA; 3 Department of Pediatrics, Geisel School of Medicine at Dartmouth, Hanover NH, USA; 4 Dartmouth-Hitchcock Weight and Wellness Center, Department of Medicine at Dartmouth-Hitchcock Medical Center, Lebanon, NH USA; 5 Department of Medicine, Geisel School of Medicine at Dartmouth, Hanover, NH USA; 6 Clinic of Preventive Medicine and Nutrition, Faculty of Medicine, University of Crete, Heraklion, Greece; 7 Department of Obstetrics and Gynecology Dartmouth Hitchcock Medical Center, Lebanon, NH USA; 8 Department of Earth Sciences, Dartmouth College, Hanover, NH USA; 9 Departments of Pediatrics and Psychiatry, Children ’s Hospital at Dartmouth, Lebanon, NH USA

**Keywords:** Toxic metals, Essential elements, Infant growth trajectories, Prenatal exposures, Growth mixture modeling, Quantile-based g-computation

## Abstract

**Background:**

Metal exposures have been associated with adverse growth in utero, but impacts on postnatal growth are not well-understood.

**Objectives:**

To examine relationships between prenatal metal exposures and infant growth trajectories in a rural U.S. pregnancy cohort.

**Methods:**

Participants included 783 mother-infant pairs in the New Hampshire Birth Cohort Study, a rural cohort of pregnant people and their children in northern New England with homes served by private unregulated drinking water. Essential and toxic element concentrations were measured in maternal toenail clippings collected at 3 weeks postpartum, reflecting exposures during pregnancy. Weight and length measures were abstracted from medical records between birth and 18 months. Weight-for-length growth trajectories were identified separately for male and female infants using growth mixture modeling. Relative risk ratios and 95% confidence intervals were estimated to evaluate associations between each element and growth trajectory assignments.

**Results:**

Four weight-for-length trajectories were identified for male and female infants: Stable-slow, Late-moderate, Stable-moderate, and Rapid growth. The Stable-slow trajectory aligned most closely with the median World Health Organization infant growth curve and was selected as the reference. Among male infants, higher maternal mercury and lead were each associated with a higher likelihood of following a growth pattern that deviated from the reference. Additionally, male infants whose mothers fell in the lowest tertile for manganese, compared with the middle tertile, were more likely to follow the Stable-moderate growth trajectory, rather than the reference. No statistically significant associations were identified for female infants.

**Discussion:**

Growth during the first 18 months of life may be accelerated in male infants exposed to higher levels of mercury or lead or to lower levels of manganese in utero. Given that accelerated growth during infancy increases risk for obesity, male infants who experience these element exposure patterns may be more susceptible to obesity later in life.

## Introduction

Abnormal growth in infancy is associated with morbidity and mortality later in life [1, 2]. For example, excess growth in infancy has been linked to increased risk of developing obesity while insufficient growth has been linked to neurodevelopmental delays in childhood [3–5]. Therefore, it is of public health importance to identify modifiable factors that influence risk of deviating from healthy growth patterns during infancy [6]. 

In utero exposures may influence postnatal growth patterns [7, 8]. The developing fetus is particularly vulnerable to stressors during this window, such as toxicant exposures and inadequate nutrition, due to immature detoxification mechanisms and a greater need for nutrients that support rapid growth [9]. Many essential and toxic elements can cross the placenta and directly affect the fetus,[10–12] possibly through impacts on epigenetic programming of energy homeostasis [13]. Some elements also influence placental function [14] which in turn may affect developmental programming and postnatal growth.

Essential and toxic elements are ubiquitous in the environment [15]. Diet is the main source of exposure to essential elements,[16] which play critical roles in normal growth and metabolism [17]. Both inadequate and excess intakes of essential elements can contribute to adverse health outcomes [18]. In contrast, toxic elements are toxic to humans even at trace levels [19]. Major sources of exposure to toxic elements include drinking water, diet, and air pollution [20]. We and others have previously reported that prenatal exposure to certain elements alters early growth [21–23]. For example, maternal toenail concentrations of lead (Pb) were associated with reduced birth weight and birth length, whereas arsenic (As) concentrations were positively associated with birth weight and length in the New Hampshire Birth Cohort Study (NHBCS). Both associations were primarily observed among female infants [21]. Maternal total urinary As was also associated with a modest increase in length during the first year of life [23]. However, prior studies have mainly focused on fetal growth [24–28] or attained anthropometry at one time point in childhood [7, 29, 30]. Previous literature suggests that heterogeneous growth patterns exist in childhood,[31] and compared with static growth measures, growth trajectories better predict future health outcomes, such as type 2 diabetes mellitus and cardiovascular diseases [31–34]. However, only a few studies have investigated associations between prenatal element exposures and growth patterns in infancy or early childhood [35–37]. To our knowledge, none of these studies have focused on rural populations in the U.S. Yet, rural areas are disproportionately impacted by both childhood obesity and many element exposures due to a higher prevalence of private unregulated drinking water and older homes [38–41]. 

To address this gap, we characterized growth trajectory patterns from birth to 18 months of age in a rural U.S. pregnancy cohort and evaluated relationships between prenatal exposure to elements and growth patterns during this window. We hypothesized that exposure to (1) low or excess levels of essential elements and (2) higher levels of toxic elements would be associated with an increased likelihood of deviating from a healthy growth pattern during infancy.

## Methods

### Study population

The NHBCS is an ongoing, prospective cohort study which was designed to examine how exposure to environmental chemicals affect maternal and child health outcomes [42]. Since 2009, mother-child pairs have enrolled from participating prenatal care clinics in the state of New Hampshire, USA. Eligibility criteria included: (1) age between 18 and 45 years, (2) receiving routine prenatal care at one of the study clinics, (3) home served by a private, unregulated water system, (4) resided in the same place since their last menstrual period and not planning to move prior to delivery, and (5) English literacy. Only singleton births were included in the study. The current study focused on mother-child pairs with element concentrations measured in maternal toenail clippings requested at approximately six weeks postpartum (Figure S1). We excluded mother-child pairs if (1) the child had a weight-for-length value lower or higher than the mean ± 4 standard deviations (*n* = 15), (2) the child had three or fewer weight or length measures available between birth and 18 months of age (*n* = 119), or (3) the mother or child was missing key covariate data (*n* = 49). The final analytic sample included 783 mother-child pairs (Figure S1). All participants provided written informed consent, and the study protocols for the NHBCS were approved by the Committee for the Protection of Human Subjects, the Institutional Review Board of Dartmouth College.

### Quantification of element concentrations

In the NHBCS, maternal toenail clippings were collected by participants at a median of 3.1 (interquartile range = 2.0, 6.1) weeks postpartum. Given that toenails generally reflect exposures that occurred approximately 7–12 months prior to collection, the sampling time points in our study reflect element exposures from periconception through mid-pregnancy [43, 44]. The toenail elements quantified in this study include aluminum [Al], antimony [Sn], As, cadmium [Cd], chromium [Cr], cobalt [Co], copper [Cu], iron [Fe], Pb, manganese [Mn], mercury [Hg], molybdenum [Mo], nickel [Ni], selenium [Se], tin [Sn], uranium [U], vanadium [V], and zinc [Zn]. Prior literature supports that the selected elements (As, Hg, Pb, Cu, Mn, Se) can be reliably measured in toenail clippings [45]. In addition to their utility as long-term biomarkers of exposure, an advantage of element concentrations in toenail clippings is that they are less susceptible to changes related to metabolic activities compared with other element biomarkers (e.g., blood or urine) [46]. NHBCS protocols related to toenail clipping collection, processing, and analysis for elements have been described in detail elsewhere [21, 47]. Briefly, study participants were provided with instructions to collect a toenail clipping from the paper envelopes separately for the great and other toes after removing any nail polish and bathing. The washed and digested toenail samples from the great toe were analyzed for element concentrations using inductively coupled plasma mass spectrometry (ICP-MS) at the Trace Element Analysis Core Laboratory (Dartmouth College, Hanover, New Hampshire, USA). The limits of detection (LOD) for toenail element concentrations were 0.023 ug/g for As, 0.031 ug/g for Hg, 0.009 ug/g for Pb, 0.105 ug/g for Cu, 0.038 ug/g for Mn, and 0.013 ug/g for Se. Concentrations below LOD were assigned a value of LOD divided by the square root of 2.[48] The coefficients of variation based on replicate analyses were 29.1% for As, 42.5% for Hg, 6.8% for Pb, 3.1% for Cu, 4.8% for Mn, and 3.9% for Se.

Given our hypothesis that both low and excess levels of essential elements (Cu, Mn, Se) would be associated with a higher risk of deviating from healthy growth in infancy, these elements were categorized into tertiles with the middle tertile serving as the reference category. Given our hypothesis that toxic elements (As, Hg, Pb) would be associated with a higher risk of deviating from healthy growth in infancy, these elements were treated continuously and were log2-transformed and scaled to reduce the influence of extreme values and facilitate comparisons across the elements, respectively. 

### Infant anthropometry measures

As described previously, infant weight and length were abstracted by trained study staff from medical records at birth and well-child visits [23, 49]. Up to 10 weight and length measurements were available from pediatric medical records reflecting the following time points: birth, 2 weeks, 1 month, 2 months, 4 months, 6 months, 9 months, 12 months, 15 months, and 18 months (median number of visits for our study sample = 9 [IQR = 8, 9]). Our primary interest was to model weight trajectories accounting for length over the first 18 months of life. However, because there is no growth standard chart for weight-for-length, we additionally calculated weight trajectories, as a weight growth standard chart does exist for this age range.

Although weight and weight-for-length z-scores can provide useful clinical information about how a child is growing compared with a growth standard, it is less useful for assessing within-person changes in growth over time and can lead to misleading results when modeled longitudinally [50]. It is therefore recommended to use raw anthropometry measures to better capture longitudinal changes in growth. Therefore, when modeling growth trajectories we used raw weight (kg) and weight-for-length (g/cm) [23, 51].

### Covariates

Information on maternal and child characteristics was collected using questionnaires administered during the prenatal and postpartum periods and through medical record abstraction. Maternal dietary information was also collected during pregnancy using a food frequency questionnaire. Potential confounders and precision variables were identified a priori based on previous literature (Figure S2), including maternal age at enrollment (years), highest educational attainment level (high school graduate or less, any college graduate, or post-graduate), marital status (married or not married), child’s in utero tobacco smoke exposure (exposed to maternal or other tobacco smoke in utero versus not exposed), maternal fish/seafood intake during pregnancy (less than once per month, once to three times per month, or once per week or more), parity during the pregnancy (nulliparous or parous), and pre-pregnancy body mass index (BMI: calculated as maternal weight prior to the pregnancy in kilograms divided by height in meters squared [kg/m^2^]). Maternal urinary arsenobetaine concentrations were measured at approximately 25.1 weeks of gestation (IQR = 23.4, 27.9). Maternal pre-pregnancy weight status (BMI < 25 or BMI ≥ 25) and urinary arsenobetaine concentration (a biomarker of fish/seafood consumption) were not included in primary models but were each considered in sensitivity analyses. Given that breastfeeding duration is associated with infant growth [52] and can be influenced by endocrine-disrupting chemicals [53], including metals [54, 55], it may lie on the causal pathway between prenatal metal exposures and infant growth. Therefore, breastfeeding duration is more likely to be a potential mediator rather than a confounder for this relationship and was not included in our analysis. Preterm birth was defined as birth before 37 weeks of gestation. Size for gestational age was categorized as small for gestational age (SGA), appropriate for gestational age (AGA), or large for gestational age (LGA) using the *gigs* R package, which applies WHO and INTERGROWTH-21st growth standards [56]. Preterm birth and SGA/LGA were reported descriptively by trajectory but are not included in primary models as these variables may lie on the causal pathway between prenatal metal exposures and postnatal growth [28, 57, 58].

### Statistical analysis

We fit a Gaussian Growth Mixture Model (GMM) [59] to examine the heterogeneity in growth patterns from birth to 18 months of age. The GMM, an extension of a mixed effects model [60], is a useful method for identifying distinct unobserved subpopulations (latent groups) that each follow an approximately homogeneous growth trajectory across time.

To estimate mean growth patterns within groups, GMMs with one to six classes were fit using a quadratic spline basis. Analyses were conducted separately for male and female infants, as early growth patterns are known to differ by biological sex assigned at birth, and we have previously reported sex differences in growth during the first year of life among NHBCS infants [23]. Knots at 3.5 and 9 months were selected to minimize the sum of squared error of spline fit [49]. Random intercepts were included in the GMM to account for repeated measures. The number of latent groups was identified using the following model fit indices: lower Bayesian Information Criterion (BIC) [61], high percentage (>80%) of participants falling into each latent class based on a mean posterior probability >0.7 [62], and class size (>5%). Any models identifying a subgroup with < 5% of participants were not considered. Each child was classified into the subgroup for which they had the highest posterior probability. Cohen’s kappa statistic was used to quantify the overlap in participants assigned to the weight versus weight-for-length trajectories [63]. 

To identify the reference group, we determined which weight trajectory subgroup most closely corresponded to the World Health Organization (WHO) growth standards charts for this age range [64]. The WHO growth standards are recommended by the U.S. Centers for Disease Control and Prevention for clinical use in the U.S. for infants and children 0 to 2 years of age [65]. These growth charts were created using longitudinal data from children in six countries throughout the world, including the U.S., who live in conditions considered optimal by the WHO (e.g., predominantly breastfed for at least four months and still breastfed at 12 months) and were constructed based on high-quality studies [65]. To determine which weight trajectory was most similar to the WHO growth standards chart, we calculated the sum of squared differences between each subgroup’s median growth measure and the median growth measure from the WHO growth standards charts at each time point between birth and 18 months of age. The group with the smallest value was selected as the reference group for all subsequent analyses. Distributions of key maternal and child characteristics were calculated for each of the estimated growth trajectory groups. 

Relative risk ratios (RRR) and 95% confidence intervals (CIs) for each element and growth trajectory were estimated assuming asymptotic normality for likelihood-based estimators. To account for the uncertainty in class assignment, we calculated these RRRs within the GMM:

$$\:RRR=\:\frac{P\left(Group\:j\right|2*Exposure)/P(Group\:j\left|Exposure\right)}{P\left(Group\:K|2*Exposure\right)/P\left(Group\:K\right|Exposure)}$$

These RRRs represent a relative risk of following each of the respective growth trajectories (Group j) for a doubling in element concentration compared with a relative risk of following the reference growth trajectory (Group K) for a doubling in element concentration [49]. For elements categorized into tertiles, the RRRs were calculated within the GMM as:

$$\:RRR=\:\frac{P\left(Group\:j\right|High\:or\:low\:tertile)/P(Group\:j\left|Middle\:tertile\right)}{P\left(Group\:K|High\:or\:low\:tertile\right)/P\left(Group\:K\right|Middle\:tertile)}$$

We performed a series of sensitivity analyses. First, to assess possible non-linear relationships between toxic elements and infant growth patterns, we categorized toxic elements into tertiles, with the low tertile serving as the reference group. Second, we assessed associations between prenatal exposure to each element and infant growth trajectories in a separate multinomial logistic regression model (i.e. using a two-stage approach rather than within the GMM), which does not account for the uncertainty in class assignment. Third, to assess the impact of knot number and location on our results, we fit a GMM with three knots that minimize the sum of squared errors. Fourth, although raw weight-for-length trajectories were our primary outcome, weight-for-length z-score has been associated with adiposity and cardiometabolic outcomes later in life [66–68]. To evaluate the associations of prenatal metal exposures with attained adiposity in infancy, we fit multivariable linear regression models between each element concentration and weight-for-length z-score at 18 months of age. In case the proportion of male or female infants in any of the identified growth group was notably smaller compared to the other growth trajectories, we examined sex-specific distributions of key characteristics in this trajectory as a *post hoc* sensitivity analysis. 

Given that maternal pre-pregnancy BMI is an important predictor of early life growth [69–71], we conducted secondary analyses in which we evaluated potential interactions between maternal pre-pregnancy BMI and each prenatal element exposure in relation to infant growth trajectories by including cross-product terms in each model. Elements commonly co-occur in the environment and may interact with each other. Therefore, their combined health impacts may differ from their individual impacts [72, 73]. In secondary analyses, we therefore also evaluated the joint impacts of prenatal element exposures on each growth trajectory membership with a two-stage approach using quantile-based g-computation and the trajectory assignments identified with GMM [74]. Quantile-based g-computation estimates the parameters of a joint marginal structural model based on generalized linear model frameworks [74]. We calculated the combined impacts of simultaneously increasing all exposures within the mixture by one quartile on the multinomial outcome (i.e., the latent growth trajectory groups) via 500 bootstrap samples. Positive and negative weights that sum to 1 in each direction were also assigned to each element within the mixture. These weights can be interpreted as the proportion of the partial impact on the outcome due to a single exposure in either direction. Given that essential and toxic elements can share common sources of exposure, such as diet and drinking water [42, 75–77], we included them together in the quantile g-computation models to reflect real-world exposure patterns.

All statistical analyses were performed using R software version 4.3.2. (R Core Team 2024) [78]. The GMM and quantile-based g-computation models were fit using the “hlme”[79] and “qgcomp” [80] packages, respectively. All models adjusted for the same list of covariates, and all tests were two-sided and used statistical significance thresholds of α = 0.05.

## Results

### Overall growth patterns of study participants during the first 18 months of life

At birth, the mean weight-for-length was 68.0 g/cm (standard deviation [SD] = 7.9 g/cm). On average, the rate of weight-for-length change increased rapidly over the first four months, declined between four and nine months, and leveled off with a mean of 135.0 g/cm (SD = 13.2) at 18 months of age (Figure S3).

### Selection of the four-class growth trajectory model for the first 18 months of life

**Fig. 1:**
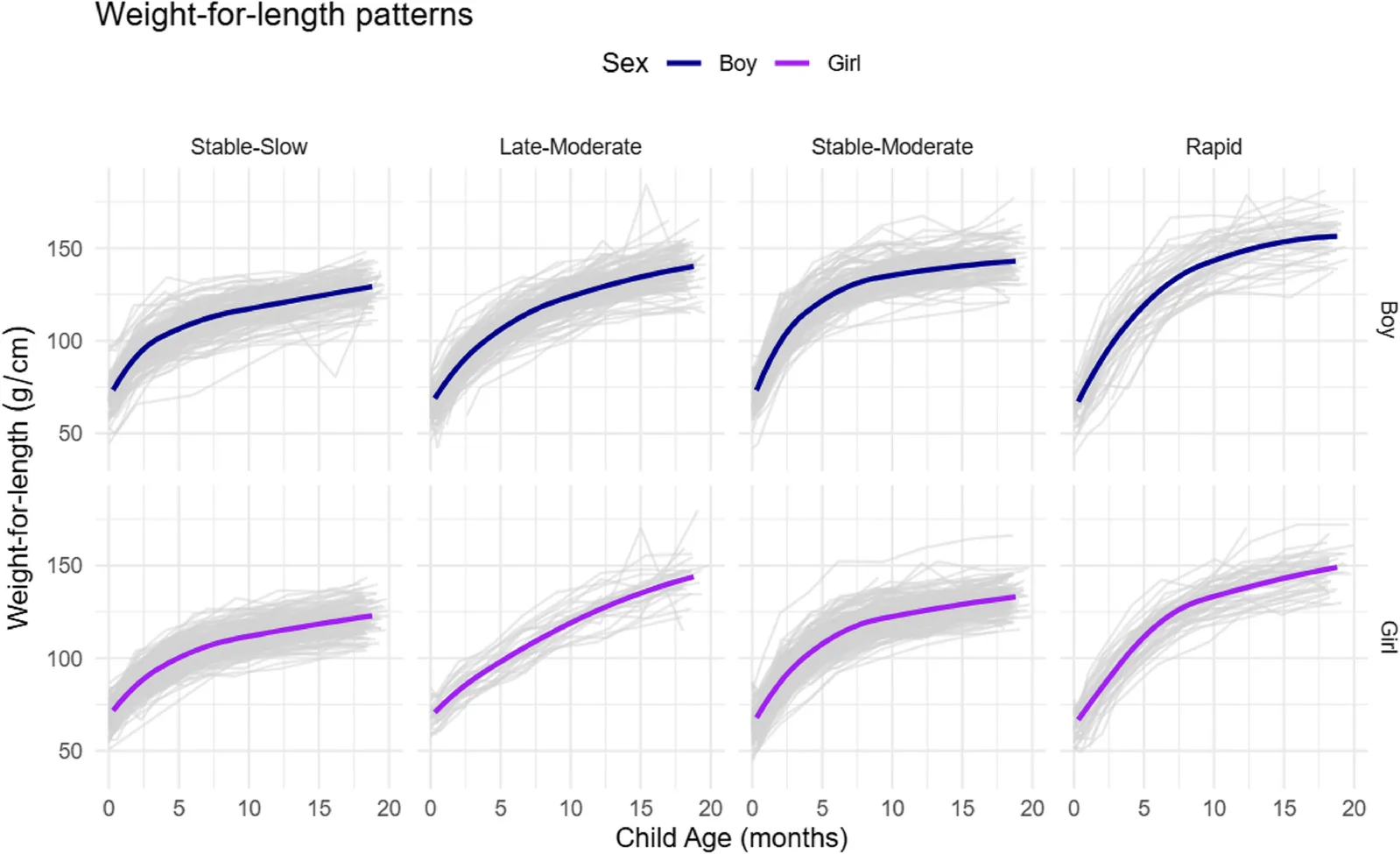
Weight-for-length growth trajectories for male and female infants. The weight-for-length (g/cm) trajectory patterns estimated by sex-stratified growth mixture modeling. Dark and light colors indicate mean or individual growth patterns, respectively, from birth to 18 months of age in the New Hampshire Birth Cohort Study

GMM fit indices for weight-for-length are shown separately for male and female infants in Table S1. In general, models with a larger number of classes (groups) had a smaller BIC. However, four trajectory groups were selected for the final model (Fig. 1), because models with a larger number of trajectories included groups with very small numbers of participants (< 5%) (Table S1). In the four-class model, approximately 80% or more of participants were classified into each group with posterior group assignment probabilities higher than 0.7. For weight, a four-trajectory model was similarly identified as optimal (Table S2). The number of observations and distributions of weight-for-length and weight by GMM trajectory class are shown in Table S3 and Table S4, respectively.

### Growth trajectory patterns during the first 18 months of life

To select the referent growth trajectory group, the median weight from the WHO growth chart [81] was subtracted from the median weight of participants in each weight trajectory at each time point (Table S5). The sum of squared differences was smallest for the slowest growth trajectory (named the Stable-slow group hereafter) and largest for the fastest growth trajectory (named the Rapid group hereafter) (Table S5). The Stable-slow group was therefore selected as the reference for all subsequent analyses.

A comparison of the WHO weight-for-age standard growth chart and each weight growth trajectory is shown in Figure S4. Overall, both male and female infants showed similar four-class growth trajectory patterns, but with different distributions across the four trajectory groups. In our labeling, “stable” indicates trajectories that showed steady growth over time, while “late” suggests trajectories that exhibited an upward deviation from the WHO growth curve later in infancy. “Moderate” and “rapid” refer to moderate or rapid excess growth, respectively, compared to the WHO reference. Specifically, “Stable-slow” represents steady growth that closely aligned with the WHO curve, “Stable-moderate” represents growth consistently above the WHO curve, “Late-moderate” represents growth showing an upward deviation from the WHO curve later in infancy, and “Rapid” describes growth that starts lower than other trajectories but accelerate above the WHO curve later in infancy.

The median weights of children in the slowest growth group (Stable-slow; 36.0% of male infants and 36.4% of female infants) were consistently lower than the median weights from the WHO growth chart across the first 18 months of life although differences were small (Table S4). Children in the second class (named the Late-moderate group hereafter; 22.8% of male infants and 6.0% of female infants) had lower median weights compared with median weights from the WHO growth chart from birth until approximately nine months, but larger median weights from nine months until 18 months of age. Children in the third class (named the Stable-moderate group hereafter; 30.6% of male infants and 42.9% of female infants) had consistently higher median weights compared with the median weight from the WHO growth chart across the first 18 months of life. Lastly, children in the fourth class (named the Rapid group; 10.6% of male infants and 14.7% of female infants) had similar median weights as the median weights from the WHO growth charts until two to four months of age, after which the median weights consistently exceeded the median weights from the WHO growth charts. This last trajectory had the highest median weight at 18 months of age for both male and female infants. Given the good agreement in participant assignments between the weight and weight-for-length trajectories (Cohen’s kappa = 0.65 in Table S6), the same group names used for the weight trajectories were also applied to each of the corresponding weight-for-length trajectories.

### Key characteristics of mother-child dyads by the growth trajectory 

Participant characteristics are shown separately by weight-for-length trajectory in Table 1. The mean maternal age at enrollment and pre-pregnancy BMI were 31.6 years and 25.8 kg/m^2^, respectively. 31% of children who followed the Stable-slow growth trajectory were first-born, compared with 52% in the Rapid growth trajectory. On average, children in the Stable-slow growth trajectory had the highest birth weight, while children in the Rapid growth trajectory had the lowest. This pattern for birth weight remained similar after accounting for key factors that are known to influence birth weight (i.e., gestational age in weeks, infant sex, and maternal parity). Distributions of weight-for-length z-score at 18 months significantly differed across the growth trajectories (*P* < 0.01), with children in the Rapid growth showing the highest z-score (mean = 1.5; standard deviation = 0.8) and those in the Stable-slow growth showing the lowest z-score (mean=-0.5; standard deviation = 0.8). While almost half (47%) of children in the Stable-slow growth trajectory were breastfed for 12 months or more, only 23% of children in the Rapid growth trajectory were breastfed for 12 months or more. Distributions of other key characteristics were similar across the different growth trajectories. Key characteristics were comparable between the NHBCS participants who were included versus excluded from the current analysis (Table S7).

**Table 1:** Descriptive characteristics of mother-child pairs by infant weight-for-length (g/cm) growth trajectory membership (*n* = 783)

Characteristic	Stable-slow (*n* = 252)	Late-moderate (*n* = 153)	Stable-moderate (*n* = 284)	Rapid (*n* = 94)	*P*
Maternal age at enrollment (years)	31.5 ± 4.2	31.5 ± 4.9	31.9 ± 4.5	31.2 ± 4.7	0.49
Maternal race					
White	245 (97.2)	147 (96.1)	280 (98.6)	92 (97.9)	0.09
Other	7 (2.8)	6 (3.9)	4 (1.4)	2 (2.1)	
Maternal education					0.71
High school graduate or less	24 (9.5)	18 (11.8)	22 (7.7)	12 (12.8)	
Any college graduate	154 (61.1)	87 (56.9)	169 (59.5)	52 (55.3)	
Post-graduate	74 (29.4)	48 (31.4)	93 (32.7)	30 (31.9)	
Maternal marital status					0.55
Married	230 (91.3)	133 (86.9)	255 (89.8)	83 (88.3)	
Not married	22 (8.7)	20 (13.1)	29 (10.2)	11 (11.7)	
Maternal parity					< 0.01
Primiparous	78 (31.0)	63 (41.2)	132 (46.5)	49 (52.1)	
Parous	174 (69.0)	90 (58.8)	152 (53.5)	45 (47.9)	
Maternal smoking in pregnancy					0.52
Never smoked and never exposed to secondhand smoke	213 (84.5)	121 (79.1)	233 (82.0)	75 (79.8)	
Former or current smoker, or exposed to secondhand smoke during pregnancy	39 (15.5)	32 (20.9)	51 (18.0)	19 (20.2)	
Maternal fish/seafood consumption					0.48
Never or less than once per month	55 (21.8)	31 (20.3)	67 (23.6)	18 (19.1)	
Once to three times per month	164 (65.1)	104 (68.0)	172 (60.6)	57 (60.6)	
Once per week or more	33 (13.1)	18 (11.8)	45 (15.8)	19 (20.2)	
Maternal pre-pregnancy BMI (kg/m^2^)	25.6 ± 5.2	25.7 ± 5.3	25.8 ± 5.5	26.5 ± 5.7	0.58
BMI < 25 kg/m^2^, N (%)	140 (55.6)	76 (49.7)	154 (54.2)	50 (53.2)	0.71
BMI≥25 kg/m^2^, N (%)	112 (44.4)	77 (50.3)	130 (45.8)	44 (46.8)	
Maternal urinary arsenobetaine level (µg/L), median (25th, 75th percentiles)	0.7 (0.1, 3.7)	0.7 (0.2, 4.6)	0.7 (0.1, 3.0)	0.9 (0.2, 5.2)	0.29
Missing	23	12	24	6	
Infant sex					< 0.01
Male	112 (44.4)	125 (81.7)	106 (37.3)	44 (46.8)	
Female	140 (55.6)	28 (18.3)	178 (62.7)	50 (53.2)	
Infant birth weight (g)	3582.5 ± 508.8	3440.1 ± 514.1	3404.0 ± 483.3	3257.9 ± 565.5	< 0.01
Infant gestational age at birth (weeks)	39.4 ± 1.2	38.9 ± 1.8	39.1 ± 1.5	38.2 ± 2.4	< 0.01
Size for gestational age					
Small for gestational age	5 (2.0)	2 (1.3)	7 (2.5)	3 (3.2)	
Appropriate for gestational age	174 (69.0)	116 (75.8)	217 (76.4)	70 (74.5)	
Large for gestational age	72 (28.6)	32 (20.9)	56 (19.7)	16 (17.0)	
Missing	1 (0.4)	3 (2.0)	4 (1.4)	5 (5.3)	
Preterm birth (gestational age < 37 weeks)	7 (2.8)	11 (7.2)	23 (8.1)	18 (19.1)	
Sex-specific birth weight-for-gestational age z-score	0.4 ± 1.1	0.2 ± 1.1	0.1 ± 1.0	0.2 ± 0.9	0.04
Missing	0	0	3	0	
Breastfeeding					< 0.01
One year of breastfeeding or more	119 (47.2)	43 (28.1)	101 (35.6)	22 (23.4)	
Less than one year of breastfeeding	108 (42.9)	87 (56.9)	149 (52.5)	61 (64.9)	
Missing	25 (9.9)	23 (15.0)	34 (12.0)	11 (11.7)	
Weight-for-length z-score at 18 months	-0.5 ± 0.8	0.6 ± 1.1	0.4 ± 0.8	1.5 ± 0.8	< 0.01

The numbers are shown as mean (standard deviation) or N (%) for continuous or categorical variables, respectively. P value estimated by analysis of variance (ANOVA) for continuous variables and chi-square test for categorical variables. Due to its right-skewed distribution, log2-transformed maternal urinary arsenobetaine levels were used for the ANOVA test

### Prenatal element exposures and growth patterns during the first 18 months of life

All six of the elements of interest were detectable in toenail clippings for ≥ 78% of the study participants (Table 2). Detailed distributions of the six elements selected for the analysis are provided in Table S8. The median and interquartile range of all elements were similar across the growth trajectory groups, although differences were observed for Hg, such that the median was slightly higher for those following the Rapid growth trajectory (0.10 µg/g) compared with the other growth trajectory memberships (0.08 µg/g; *P* = 0.11).

**Table 2:** Maternal toenail element concentrations postpartum by infant weight-for-length (g/cm) growth trajectory membership (*n* = 783)

	Stable-slow (*n* = 252)		Late-moderate (*n* = 153)		Stable-moderate (*n* = 284)		Rapid (*n* = 94)		*P*
Element	Detection frequency (%)	Median (IQR)	Detection frequency (%)	Median (IQR)	Detection frequency (%)	Median (IQR)	Detection frequency (%)	Median (IQR)	
As, µg/g	88	0.05 (0.04, 0.09)	90	0.05 (0.03, 0.08)	90	0.05 (0.04, 0.08)	85	0.06 (0.04, 0.09)	0.83
Hg, µg/g	78	0.08 (0.03, 0.14)	80	0.08 (0.04, 0.15)	79	0.08 (0.04, 0.19)	89	0.10 (0.05, 0.18)	0.11
Pb, µg/g	99	0.12 (0.06, 0.21)	99	0.10 (0.05, 0.21)	99	0.10 (0.05, 0.22)	98	0.12 (0.05, 0.27)	0.31
Cu, µg/g	90	3.96 (3.30, 4.89)	88	3.85 (3.21, 4.41)	89	3.82 (3.24, 4.78)	91	3.85 (3.36, 4.48)	0.65
Mn, µg/g	93	0.34 (0.17, 0.63)	89	0.32 (0.16, 0.63)	87	0.27 (0.14, 0.59)	93	0.32 (0.18, 0.78)	0.18
Se, µg/g	100	1.00 (0.91, 1.09)	100	1.00 (0.92, 1.11)	100	1.00 (0.91, 1.09)	100	0.99 (0.90, 1.09)	0.85

IQR indicates 25th, 75th percentiles. P value was estimated by analysis of variance (ANOVA) for log2-transformed element concentrations

*Abbreviations*: *As *Arsenic, *Hg* Mercury, *Pb* Lead, *Cu* Copper, *Mn* Manganese, *Se* Selenium

**Fig. 2:**
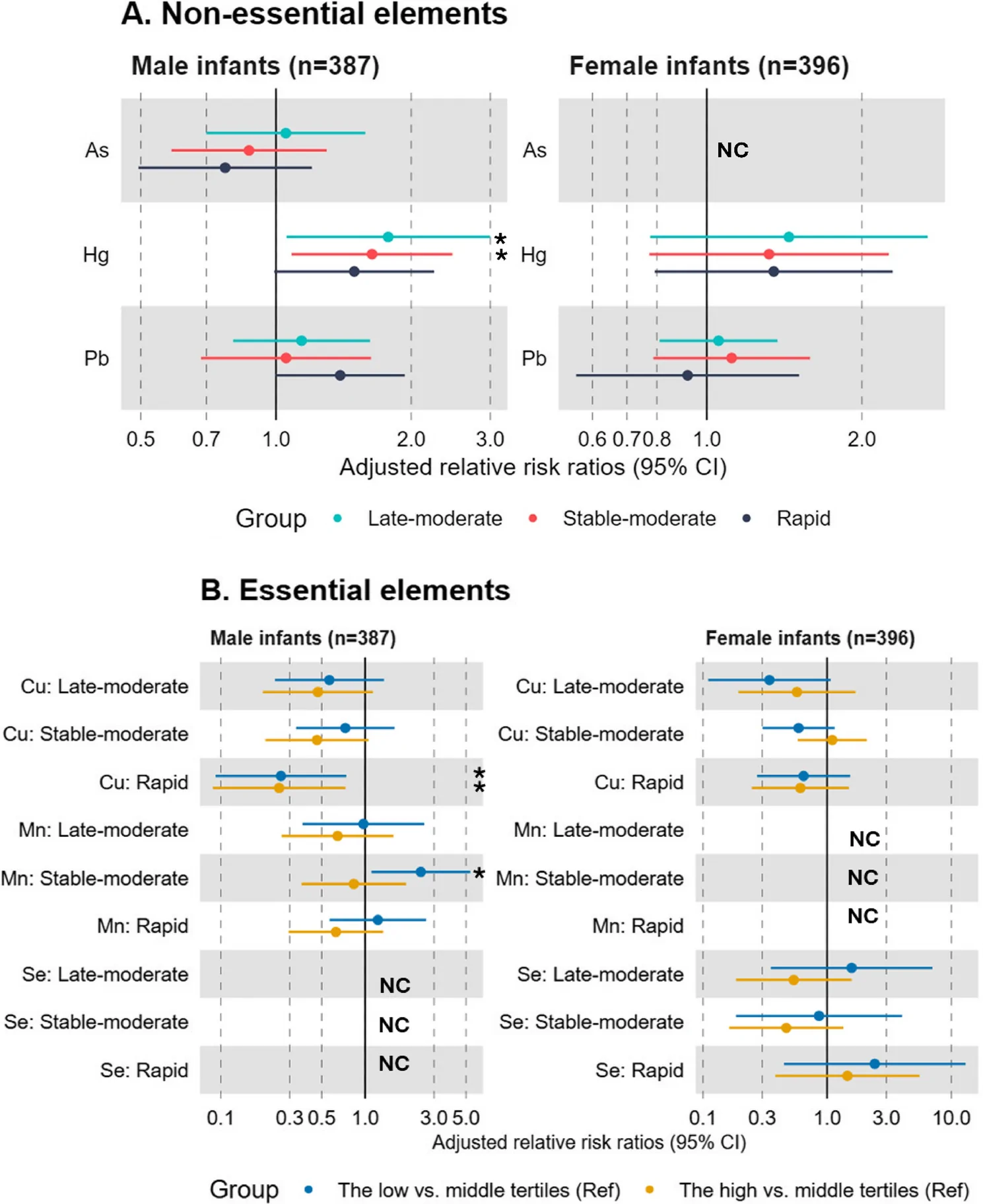
Relative risk ratios (95% CI) for weight-for-length (g/cm) growth trajectory patterns by maternal toenail element concentrations in the New Hampshire birth cohort study (*n* = 783). For panel **A**, relative risk ratios were estimated comparing each weight-for-length (g/cm) growth trajectory group to the reference group (stable-slow growth) for a doubling in maternal toenail element concentrations since the hypothesis was that high values were harmful. For panel **B**, relative risk ratios were estimated comparing each weight-for-length (g/cm) growth trajectory group to the reference group (stable-slow growth) for the low or high tertile of maternal toenail element concentrations compared to the middle tertile since either low or high values are potentially harmful. Statistically significant results (P-value < 0.05) are indicated with asterisks. The selenium model did not converge for male infants. The arsenic and manganese models did not converge for female infants. All models were adjusted for maternal age at enrollment, educational attainment level, marital status, infant in utero tobacco smoke exposure, fish and seafood consumption during pregnancy, pre-pregnancy body mass index (kg/m2), and parity. Abbreviations: *As* Arsenic, *Hg* Mercury, *Pb* Lead, *Cu* Copper, *Mn* Manganese, *NC* Model did not converge, *Se* Selenium

Unadjusted and covariate-adjusted associations between prenatal element exposures and each weight-for-length growth pattern are presented as RRR separately by sex, with the Stable-slow group modeled as the reference. Because toxic element concentrations were log_2_-transformed, results can be interpreted as the difference in risk of following a given growth trajectory, as compared with the reference, for a doubling in the toxic element concentration (Fig. 2 and Table S9). Overall, the magnitudes and directions of the RRR estimates were consistent before and after adjusting for selected covariates. Male infants whose mothers had higher toenail Pb concentrations had an increased likelihood of following the Rapid growth trajectory (RRR = 1.39; 95% CI = 1.00, 1.94) as compared to the Stable-slow growth trajectory. Male infants whose mothers had higher toenail Hg concentrations had an increased RRR of following the Late-moderate (1.78; 95% CI = 1.06, 3.00), Stable-moderate (1.64; 95% CI = 1.08, 2.47), and Rapid growth trajectories (1.49; 95% CI = 0.99, 2.25) as compared to the Stable-slow growth trajectory. Among female infants, associations for Hg and Pb were null. Associations for As were either not statistically significant (males) or models could not converge (females). We found no evidence of non-linear associations between any of the toxic elements and growth trajectories (Fig. 2 and Table S10).

Potential non-linear associations were observed between some of the essential element concentrations and growth trajectories (Fig. 2 and Table S11). Male infants whose mothers had toenail Cu concentrations falling in the low or high tertile, rather than the middle tertile, were less likely to follow the Rapid growth trajectory (RRR = 0.26; 95% CI = 0.09, 0.74 for the low tertile; RRR = 0.25; 95% CI = 0.09, 0.73 for the high tertile), compared with the Stable-slow growth trajectory. Similar patterns were observed among female infants, but associations were not statistically significant. Male infants whose mothers had Mn concentrations falling in the low rather than middle tertile were more likely to follow the Stable-moderate growth trajectory compared with the Stable-slow growth trajectory (RRR = 2.43; 95% CI = 1.11, 5.35). Associations between Mn and growth trajectories could not be examined among female infants due to a lack of model convergence. Associations between Se tertiles and growth trajectories were largely non-significant among female infants and could not be evaluated among male infants due to non convergence.

### Sensitivity and secondary analyses

Results were similar when the RRR was estimated in separate multinomial logistic regression models, except for Pb (Table S12). When using this two-stage approach, a doubling in Pb was associated with a lower likelihood of following the Late-moderate (RRR = 0.79; 95% CI = 0.60, 1.03) and Stable-moderate growth patterns (RRR = 0.77; 95% CI = 0.58, 1.02) as compared with the referent Stable-slow growth, although associations were not statistically significant. In the comparison between the two- and three-knot GMM models, the fit indices consistently supported four latent classes for male infants (Table S13). However, three latent classes were selected for female infants due to the small size of one of the identified classes. Nonetheless, the class assignments between the two- and three-knot models were highly consistent, with a Cohen’s kappa of 0.74 (95% CI = 0.70, 0.78), indicating substantial agreement (Table S14). Among male infants, a doubling of Hg was associated with a higher weight-for-length z-score at 18 months (β = 0.19; 95% CI = 0.04, 0.33), which is consistent with the observed associations between Hg and likelihood of deviating from the reference growth pattern in our main analysis (Table S15). While no association was found between element concentrations and growth trajectories among female infants in our main analysis, those in the lowest tertile of Mn had a higher weight-for-length z-score at 18 months (β = 0.38; 95% CI = 0.09, 0.68; Table S16). The proportion of female infants in the Late-moderate growth group was notably smaller than that of male infants. Within this group, 71.4% of female infants (vs. 45.6% of male infants) were born to mothers with pre-pregnancy overweight or obesity (Table S17). Female infants also tended to have higher sex-specific birth weight-for-gestational age z-scores (mean ± SD = 0.63 ± 1.05 vs. 0.07 ± 1.08 for males) and higher weight-for-length z-scores at 18 months (1.25 ± 1.76 vs. 0.41 ± 0.85 for males). Cross-product interaction terms for each element and maternal pre-pregnancy weight status were not statistically significant.

Statistically significant correlations for element pairs ranged from 0.12 (between Cu and Mn) to 0.41 (between Mn and Pb; Figure S5). When using a two-stage quantile-based g-computation model, none of the joint associations between the element mixtures and growth trajectory groups reached statistical significance. A simultaneous one quartile increase in all elements was associated with a higher risk of following the Late-moderate (RR = 1.28; 95% CI = 0.76, 2.16) or Rapid growth pattern (RR = 1.74; 95% CI = 0.85, 3.58) in male infants only (Table 3). Hg was the main contributor to these positive associations (weight = 0.50 and 0.58 for the Late-moderate and Rapid growth trajectories, respectively). Among female infants, a simultaneous one quartile increase in all elements was associated with a higher risk of following the Rapid growth pattern (RR = 1.23; 95% CI = 0.58, 2.61), with Pb as the main contributor (weight = 0.49; Table 4).

**Table 3:** Risk ratios (95% CI) for weight-for-length growth trajectory patterns for a simultaneous quartile increase in the element mixture among male infants, estimated by quantile-based g-computation (*n* = 387)

Exposure	Late-moderate		Stable-moderate		Rapid	
	Mixture RR (95% CI)		Mixture RR (95% CI)		Mixture RR (95% CI)	
	1.28 (0.76, 2.16)		1.04 (0.62, 1.74)		1.74 (0.85, 3.58)	
	Element Weights		Element Weights		Element Weights	
	Positive	Negative	Positive	Negative	Positive	Negative
As	0.29		0.18		0.27	
Hg	0.50		0.55		0.58	
Pb		1.00		0.46		0.92
Cu	0.02		0.27		0.13	
Mn	< 0.01			0.42	0.02	
Se	0.19			0.12		0.08

For all models, the reference growth trajectory pattern was the Stable-slow growth. Adjusted for maternal age at enrollment, educational attainment level, marital status, infant in utero tobacco smoke exposure, fish and seafood consumption during pregnancy, pre-pregnancy body mass index (kg/m^2^), and parity

*Abbreviations*: *As* Arsenic, *Hg* Mercury, *Pb* Lead, *Cu* Copper, *Mn* Manganese, *Se* Selenium

**Table 4:** Risk ratios (95% CI) for weight-for-length growth trajectory patterns for a simultaneous quartile increase in the element mixture among female infants, estimated by quantile-based g-computation (*n* = 396)

Exposure	Late-moderate		Stable-moderate		Rapid	
	Mixture RR (95% CI)		Mixture RR (95% CI)		Mixture RR (95% CI)	
	1.01 (0.45, 2.30)		0.81 (0.52, 1.28)		1.23 (0.58, 2.61)	
	Element Weights		Element Weights		Element Weights	
	Positive	Negative	Positive	Negative	Positive	Negative
As		0.39		0.31		0.01
Hg		0.37		0.06		0.03
Pb		0.24	0.32		0.49	
Cu	0.10			0.56		0.96
Mn	0.20			0.07	0.21	
Se	0.70		0.68		0.31	

For all models, the reference growth trajectory pattern was the Stable-slow growth. Adjusted for maternal age at enrollment, educational attainment level, marital status, infant in utero tobacco smoke exposure, fish and seafood consumption during pregnancy, pre-pregnancy body mass index (kg/m^2^), and parity

*Abbreviations*: *As* Arsenic, *Hg* Mercury, *Pb* Lead, *Cu* Copper, *Mn* manganese, *Se* Selenium

## Discussion

In this well-characterized rural U.S. pregnancy cohort, we evaluated associations between maternal toenail element concentrations reflecting exposures during pregnancy and infant growth trajectories from birth to 18 months of age. Using GMM, we identified four infant growth patterns for both male and female infants. These growth patterns included a Stable-slow, Late-moderate, Stable-moderate, and Rapid growth group, with the Stable-slow group deviating least from the WHO standard growth chart for this age. Among male but not female infants, higher maternal Hg and Pb concentrations were associated with increased risk of deviating from the referent growth trajectory and potential non-linear associations were observed for maternal Cu and Mn in relation to growth patterns. Many of the results were similar when using an environmental mixture modeling approach (quantile-based g-computation), although they did not reach statistical significance.

A small number of prior studies in China and Japan have investigated associations between prenatal exposure to elements and early life growth trajectories [35–37]. For example, in the Japan Environment and Children’s Study associations between maternal blood levels of Pb, Cd, Hg, Se, and Mn during pregnancy were assessed in relation to child growth trajectories during the first 3 years of life [35], and a study in Jiangsu province, China assessed relationships between maternal urinary levels of 17 elements, including As, Cu, Hg, and Pb, measured in both early and late pregnancy, and growth trajectories from 1 to 12 months of age [36]. In contrast with our approach, these prior studies modeled trajectories using standardized weight [35], length [36], or BMI scores [37] and did not select their reference based on the WHO growth charts. Thus, results are not directly comparable. However, it should be noted that Pb and Hg were associated with deviations from a reference growth trajectory in the first three years of life in the study by Taniguchi et al., although the directionality differed from our findings [35]. For example, higher maternal Pb levels were associated with increased odds of infants following Group I (very small to small) or Group II (moderately small), compared to the reference trajectory (Group III: moderately small to moderately large). Additionally, higher maternal Hg levels were linked to decreased odds of following Group IV (moderately large to normal) and Group V (moderately large to large), compared to the reference trajectory (Group III: moderately small to moderately large). In contrast, we found that higher maternal Pb and Hg levels were each associated with a higher likelihood of following growth patterns suggesting excess growth as compared with the reference trajectory (the Stable-slow growth). Recently, Liu et al. [37] also examined associations of multiple elements measured in maternal serum in the first trimester of pregnancy, including Cu and Pb, with child growth trajectories from birth until 12 months of age in a prospective cohort study in Shenzhen, China. Similar to our study, they applied GMMs and identified four growth trajectories. However, they modeled BMI-for-age z-score. In single-element models, maternal serum Cu levels were associated with a lower likelihood of following a more rapid BMI z-score trajectory over childhood. In contrast, we observed a non-linear relationship between maternal toenail Cu concentrations and a likelihood of following the Rapid growth trajectory. To our knowledge possible non-linear relationships between Cu and early growth patterns have not previously been assessed. 

In the current study, higher maternal toenail Hg concentrations were associated with an increased risk of male infants following a growth pattern that deviates from a growth trajectory most consistent with the WHO standard. The general population is primarily exposed to methyl Hg, the primary form reflected in toenails, through diet from fish consumption [45, 75]. Although Hg is mainly known for its neurotoxic effects [82], growing evidence suggests that Hg may also contribute to increased risk for obesity [83–86]. For example, a study in the Boston Birth Cohort [87] reported that children whose mothers had higher red blood cell Hg concentrations 1–3 days after delivery were at increased risk of overweight or obesity from 2 to 15 years of age. Similarly, a study in Korea [88] reported that higher cord blood Hg levels were associated with a rapid increasing trend of weight from birth to 44 months of age. However, other studies have reported inverse [35, 89, 90] or null associations [91] between prenatal Hg exposure and early growth measures, possibly due to differences in biomarkers, levels of exposure, or assessment and timing of anthropometry measures. Although the biological mechanisms underlying prenatal Hg impacts on early life growth are not fully understood, there is evidence that this element may dysregulate leptin, which is known to decrease food intake and body weight, or disrupt energy homeostasis by altering PPAR-α or PPAR-γ expression [92, 93]. Although the reason we observed sex-specific associations between Hg and infant growth patterns is unknown for humans, male rats are known to excrete Hg from the body more slowly than females [94, 95], and thus, males may be more sensitive to the adverse effects of Hg. However, this will need to be explored further. 

In primary models, higher maternal toenail Pb concentrations were associated with an increased likelihood of following the Rapid Growth pattern among male infants. Surprisingly, when using a two-stage approach that did not account for the uncertainty of growth trajectory assignment, the direction of association for Pb and rapid growth reversed. One possible explanation for the difference between the one and two-step approaches may be that individuals with certain levels of Pb exposure had relatively weak classification probabilities (i.e., near 0.5). The classification uncertainty is accounted for in the one-step approach, whereas the two-step approach ignores this uncertainty and is therefore more likely to misclassify study participants, especially when probabilities are borderline. This finding emphasizes the importance of selecting appropriate statistical approaches when analyzing growth trajectories identified by GMM. Air, drinking water, food, and indoor dust are the primary sources of Pb exposure for the general population [45, 75]. In our study population, drinking water may represent an important source, possibly related to older housing and associated plumbing [96]. Pb is known for its neurotoxicity [97] and prior animal and epidemiological studies have also reported potential impacts on child growth in a sex-dependent manner. For example, Leasure et al. [98] found that in utero exposure to Pb increased weaning weight among male mice, but not female mice, consistent with our findings. Similarly, Faulk et al. [99] observed that male mice exposed to Pb prior to mating through lactation had higher body weights than unexposed mice. More recently, a study in the Boston Birth Cohort [100] reported that children who had been exposed prenatally to higher levels of Pb during pregnancy had an increased risk of overweight or obesity at eight years of age. In contrast, they did not observe differences by sex. Other studies have reported inverse [101, 104, 107–109] or null associations [110–112] between prenatal Pb exposure and excessive growth in early life. Notably, most of the studies reporting inverse associations between prenatal exposure to Pb and excessive early growth have been conducted in Mexico [101, 104, 107–109]. Possible reasons for this discrepancy include different biomarkers, different range of Pb concentrations, or different statistical approaches. It would be important to confirm our findings in future epidemiological studies that could examine the associations of maternal toenail Pb concentrations spanning different ranges from the current study with infant growth trajectories.

There are several plausible mechanisms by which prenatal exposure to Pb may contribute to growth patterns postnatally. Maternal Pb can cross the placenta [113] and induce epigenetic reprogramming [114]. A study using a rat model [115] reported that Pb exposure induced higher expression of Dnmt3a, a DNA methyltransferase expressed during embryogenesis, in the hippocampus among males animals only [116]. This was associated with a reduced gestational age at birth, which in turn has been associated with rapid catch up growth [117, 118]. Impacts on food intake or activity levels may also play a role and could explain some of the observed sex differences in our study, as experimental studies using mouse models have reported early onset of overeating [99] and reduced overall activity [98] among male mice exposed in utero to Pb. Interestingly, in female animals there was a delay before changes in food intake occurred [99]. Specifically, food intake increased by 6 months and plateaued by 9 months of age among male mice, whereas this trend appeared at 9 months of age among female mice. Thus, future epidemiological studies that evaluate Pb relationships with food intake or physical activity and assess growth patterns through late childhood for both male and female children would be informative.

In the general population, Mn exposure primarily occurs through the consumption of food and water [45]. Some populations, particularly users of private wells in the U.S., may be at risk of higher Mn exposure [77]. Prior studies have reported U-shaped associations between Mn and adiposity [119] and metabolic disorders [120, 121]. Consistent with this hypothesis, we observed that male infants in the lowest tertile for maternal Mn were more likely to follow the Stable-moderate growth pattern (rather than the Stable-slow growth pattern) as compared with those in the middle tertile, which suggests that low levels of Mn in utero may contribute to more accelerated growth in the postnatal window. Manganese is an essential trace element that serves as a cofactor for a range of enzymes involved in energy metabolism, antioxidant defense, neurotransmitter synthesis, and reproductive hormone regulation [122]. Low Mn levels in early life may contribute to excess growth as observed in our study due to metabolic dysregulation induced by impaired insulin secretion [123, 124] or increased oxidative stress [125, 126]. However, this finding should be interpreted with caution, as in this relatively healthy U.S. population participants in the low tertile of toenail Mn concentrations do not necessarily have clinical Mn deficiency.

Notably, we did not observe a similar association for the high tertile of Mn, and we did not observe any associations between maternal toenail Mn and infant growth trajectories among female infants. Mn absorption efficiency is lower among male infants compared with female infants [127, 128], which could explain these sex differences. Prior studies have also reported sex-specific impacts of prenatal exposure to Mn on postnatal growth. For example, a study in China which identified six trajectories based on length for age z-score up to 12 months of age [36] found that early pregnancy exposure to Mn was associated with a higher likelihood of following an excess growth trajectory among male, but not female, infants. Another study in China [129] reported inverse associations between cord blood Mn concentrations and BMI z-score were among male and female infants at 1 and 2 years of age, respectively. The potential sex-specific impacts of Mn on infant adiposity should be replicated in other epidemiological studies.

Cu is also an essential element [130], with the main sources of exposure including air, food, water, and supplements, with diet likely the major source in this population [131]. We similarly observed non-linear associations between this element and infant growth. However, the patterns of associations were opposite from what we expected, with an inverse U-shaped association between Cu and the risk of deviating from the reference (Stable-slow) growth trajectory. Specifically, male infants whose mothers were in the low or high tertile of Cu were less likely to follow the Rapid growth trajectory, compared with those in the middle tertile. These associations are not consistent with previous cross-sectional studies, which reported positive associations between serum Cu levels and risk of overweight/obesity in childhood or adulthood [132, 133]. This may be due to the different exposure windows assessed. It is also possible that these prior cross-sectional findings are driven by reverse causality, which is not a plausible explanation for results from our prospective study. A possible biological explanation for the association between the low tertile of Cu and a lower likelihood of following the Rapid growth trajectory, compared to the Stable-slow growth, is that low copper levels may suppress insulin-like growth factor-1 production [134] or impair appetite regulation [135], which may limit energy intake and anabolic growth processes. Similarly, the inverse association observed for the high tertile of Cu may be explained by Cu’s competitive interactions with other essential micronutrients such as Fe and Zn, which are critical for early growth [136, 137]. In excess, Cu may reduce the bioavailability or utilization of these nutrients, which may paradoxically protect against an infant following a Rapid growth pattern in early life.

Given that all of the participants in our study sample had a private unregulated water source at their home, they are at increased risk of As exposure, which is a major concern in this region [138, 139]. The general population is mainly exposed to inorganic As through the ingestion of contaminated drinking water and food [42, 45, 76]. Arsenic can cross the placenta and disrupt metabolic programming in the fetus [140–142]. Prior studies both in this cohort and other populations have reported associations between As exposure and reduced fetal growth, which is a risk factor for catch-up growth during infancy [143, 144]. However, associations between prenatal As exposure and infant growth trajectories were null in the current study. Although several animal studies have demonstrated that in utero exposure to As may lead to an increase in weight gain in the early postnatal period or higher fat mass in young adulthood [140, 145, 146], other studies reported reduced body weight [147] or no associations [148, 149]. Notably, many of the prior epidemiological studies assessing relationships between prenatal As exposure and child growth were conducted in Bangladesh, where malnourishment is prevalent; these studies observed reduced growth or stunting in relation to As [110, 150, 151]. However, a study in Spain [152] reported a null association between prenatal As exposure and weight gain and obesity at 7 years of age similar to our null findings for growth patterns in infancy. It is therefore possible that As may have differential effects on growth depending on whether the population is in an obesogenic environment. Previously, we reported that higher in utero As was associated with a longer length during the first year of life in this cohort, but did not observe significant associations with weight gain or change in weight-for-length during this early window [23]. Results from the current study suggest that prenatal As also does not influence weight-for-length later in infancy. However, we cannot rule out the possibility that there may be delayed impacts on adiposity that become apparent later in childhood, which can be assessed in future studies as these children continue to be followed up.

Weight-for-length z-score has previously been associated with increased adiposity and potential cardiometabolic risk later in life [66–68]. To further explore potential implications of the identified growth trajectories, we therefore examined the distribution of weight-for-length z-scores at 18 months across the different growth trajectories. Children in the Rapid growth trajectory showed the highest weight-for-length z-score at 18 months (mean = 1.5; standard deviation = 0.8), followed by those in the Late-moderate (mean = 0.6; standard deviation = 1.1), then Stable-moderate (mean = 0.4; standard deviation = 0.8), then Stable-slow (reference group; mean=-0.5; standard deviation = 0.8) trajectories. Although weight-for-length z-scores in the Late-moderate and Stable-moderate were not as high as that observed for the Rapid growth group, the clinical implications of these trajectories warrant further consideration and can be examined in more detail in the future as these participants are followed further out into childhood. This will be an important future direction for this ongoing cohort study.

The present study has some limitations that are important to acknowledge. First, this study used element concentrations measured in maternal toenail samples collected at median 3.1 weeks postpartum, which reflects exposures over the past seven to 12 months [43, 44]. While these measures are excellent long-term biomarkers of elements exposure before or during pregnancy, the specific windows of exposure during the prenatal period that are most sensitive to these elements cannot be discerned. In addition, although prior literature has reported relatively strong correlations between toenail and blood concentrations for Hg (*r* = 0.78) and Se (*r* = 0.91), other elements generally show low to moderate correlations [43, 44, 153]. Differences across biomarkers may reflect not only differences in measurement reliability but also differences in the exposure window captured, the chemical forms of the element, and tissue-specific kinetics. This variability may partly explain discrepancies between our findings and previous studies that assessed element exposures using other biomarkers. Second, we focused on postnatal growth patterns from birth to 18 months of age, yet there may be impacts of these elements on growth patterns later in childhood. Although this could not be assessed in the current study, there will be future opportunities to assess element impacts on growth trajectories later in childhood, since this is an ongoing longitudinal study. Third, although the current study is one of the largest to examine metal exposures in relation to growth trajectories in early life, some of our models did not converge—a limitation that is not uncommon in highly complex models including growth mixture modeling [49]. Non-convergence may be partly attributable to small sample size in certain latent classes, such as the Late-moderate group among female infants, potentially limiting statistical power. Despite this, we were still able to characterize relationships between a number of metal exposures during the prenatal period and distinct early-life growth patterns. Lastly, to our knowledge, there are no nationwide pregnancy cohort studies measuring element exposures using toenail samples. Therefore, direct comparisons of toenail element concentrations to the general population were challenging in this study.

Our study also had many strengths. To our knowledge, this was one of the first studies to evaluate relationships between prenatal element exposures and infant growth trajectories in the rural U.S. We assessed multiple elements, including both essential and toxic elements, and considered them individually and as a complex mixture. We also considered potential non-linear associations between each element and infant growth and adjusted for a comprehensive list of covariates, such as fish intake, which was not considered in similar studies that assessed the impact of Hg exposure on child growth [35, 36]. Because toenail element concentrations reflect long-term exposures, they are less sensitive to day-to-day variability compared with other element biomarkers, such as urinary and blood element concentrations. Compared with prior similar studies using standardized growth measures [35–37] for growth trajectory modeling, we modeled trajectories of raw weight-for-length, which is more appropriate for examining longitudinal changes in adiposity over time. Additionally, we estimated RRRs within GMM to account for uncertainty in the trajectory assignments. Finally, a major strength is our focus on a rural population that is especially susceptible to element exposures due to their reliance on private unregulated drinking water. Despite being more susceptible to certain element exposures [38, 39] and childhood obesity [40, 41]. rural populations have been historically underrepresented in U.S. biomedical research [154]. This is therefore an important population in which to address this question.

In conclusion, in a rural U.S. pregnancy cohort, we found that male infants exposed to higher levels of Hg and Pb and lower levels of Mn in utero were more likely to follow growth patterns deviating from the WHO growth standard curves during the first 18 months of life. In contrast, male infants whose mothers had Cu concentrations falling in the low or high tertile were less likely to follow the Rapid growth trajectory. Further studies are needed to assess whether these findings are generalizable to other populations and whether the sex-specific effects that we observed may persist beyond infancy.

## Supplementary Information

Supplementary Material 1.

## Data Availability

The data that support the findings of this study are available from the New Hampshire Birth Cohort Study (NHBCS). The data will be available upon reasonable request and with the permission of NHBCS.
