# Supplementary material for: Prenatal exposure to essential and toxic elements in relation to infant growth trajectories
Source: Environ Health. 2026 Jan 9;25:17. doi: 10.1186/s12940-025-01252-w (PMC12930931; doi:10.1186/s12940-025-01252-w)
Supplement: Supplementary file 1 — Supplementary Material 1. [file 12940_2025_1252_MOESM1_ESM.pdf]

Supplemental Material

Prenatal exposure to essential and toxic elements in relation to infant growth trajectories

Gyeyoon Yim, Brianna C Heggeseth, Diane Gilbert-Diamond, Janet L Peacock, Katerina Margetaki, Emily R Baker, Thomas J Palys, Brian P Jackson, Juliette C Madan, Megan E Romano, Margaret R Karagas, Caitlin G Howe

Table of contents:
Figure S1. Selection of the study population for weight-for-length (g/cm) outcomes
Figure S2. Directed Acyclic Graph for covariates adjusted for in main analysis
Figure S3. Weight (A) and Weight-for-Length (B) measures at each time point for each participant during the first 18 months of life
Table S1. Growth mixture model fit indices for infant weight-for-length (g/cm) in the New Hampshire Birth Cohort Study
Table S2. Growth mixture model fit indices for infant weight (kg) in the New Hampshire Birth Cohort Study
Table S3. Number of observations and distribution of weight-for-length (g/cm) by infant's growth mixture modeling (GMM) trajectory class
Table S4. Number of observations and distribution of weight (kg) by infant's growth mixture modeling (GMM) trajectory class
Table S5. Selection of a reference weight (kg) growth trajectory group using sum of squares
Figure S4. Weight (kg) growth trajectories for male and female infants
Table S6. Overlap between infant growth trajectory memberships estimated using weight (kg; n=787) versus weight-for-length (g/cm; n=783)
Table S7. Descriptive characteristics of mother-child pairs excluded from analysis (n=183)
Table S8. Detailed distributions of selected elements in the New Hampshire Birth Cohort Study (n=783)
Table S9. Relative risk ratios (95% CI) for weight-for-length growth trajectory patterns by maternal toenail toxic element concentrations (continuous) in the New Hampshire Birth Cohort Study (n=783)
Table S10. Relative risk ratios (95% CI) for weight-for-length growth trajectory patterns by maternal toenail toxic element concentrations (tertiles) in the New Hampshire Birth Cohort Study (n=783)
Table S11. Relative risk ratios (95% CI) for weight-for-length growth trajectory patterns by maternal toenail essential element concentrations (tertiles) in the New Hampshire Birth Cohort Study (n=783)
Table S12. Relative risk ratios (95% CI) for weight-for-length growth trajectory patterns by maternal toenail toxic metal (continuous) and essential metal concentrations (tertile) in the New Hampshire Birth Cohort Study, using a two-stage approach (n=783)
Table S13. Growth mixture model fit indices for infant weight-for-length (g/cm) in the New Hampshire Birth Cohort Study, with 3 knots
Table S14. Overlap between infant growth trajectory memberships estimated using weight-for-length (g/cm), comparing 2 knots and 3 knots (n=783)

Table S15. Difference (95% confidence interval) in weight-for-length z-score at 18 months of age by maternal toenail toxic element concentrations (continuous) in the New Hampshire Birth Cohort Study (n=783)
Table S16. Difference (95% confidence interval) in weight-for-length z-score at 18 months of age by maternal toenail essential element concentrations (tertiles) in the New Hampshire Birth Cohort Study (n=783)
Table S17. Descriptive characteristics of mother-child pairs among those assigned to the Late-moderate growth, stratified by sex (n=153)
Figure S5. Pearson correlations between toenail element pairs (n=783)

Figure S1. Selection of the study population for weight-for-length (g/cm) outcomes

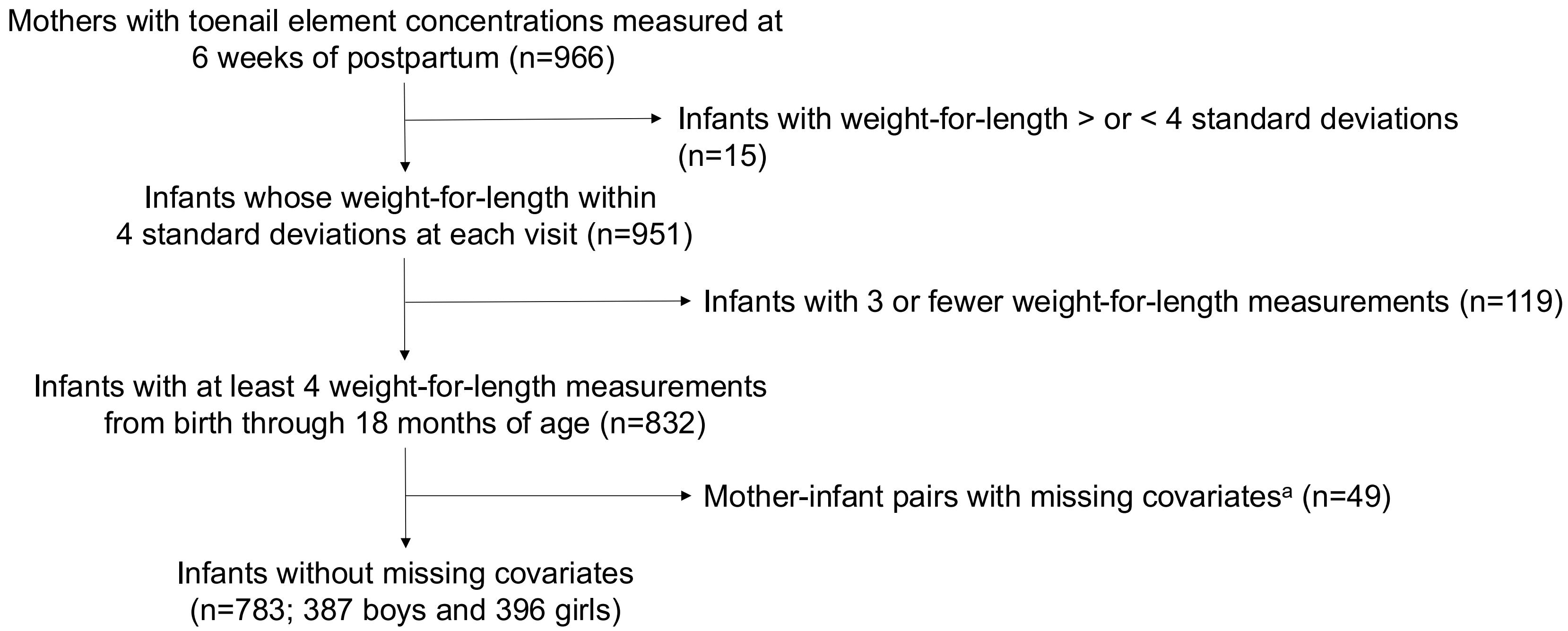

[a]Covariates included maternal age at enrollment, educational attainment level, marital status, tobacco smoke exposure during pregnancy, fish and seafood consumption during pregnancy, pre-pregnancy body mass index (kg/m^2), and parity.

Figure S2. Directed Acyclic Graph for covariates adjusted for in main analysis

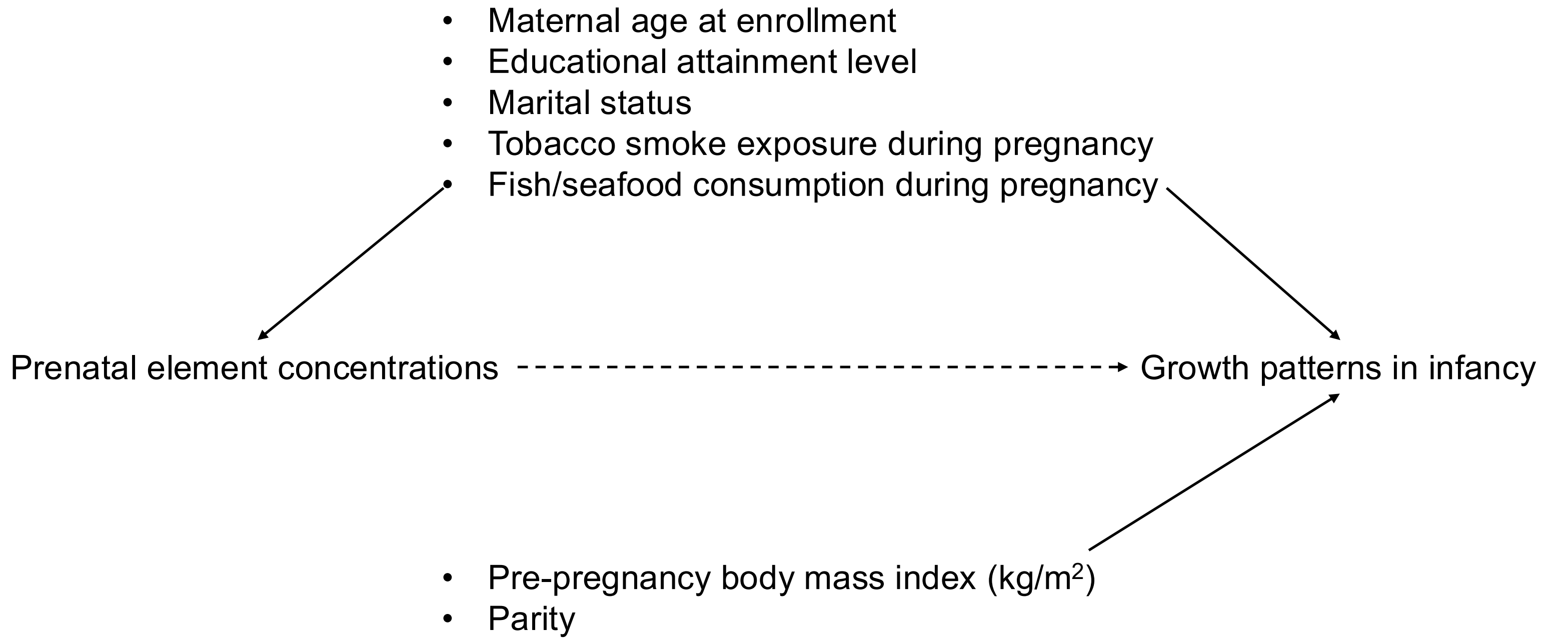

Figure S3. Weight (A) and Weight-for-Length (B) measures at each time point for each participant during the first 18 months of life

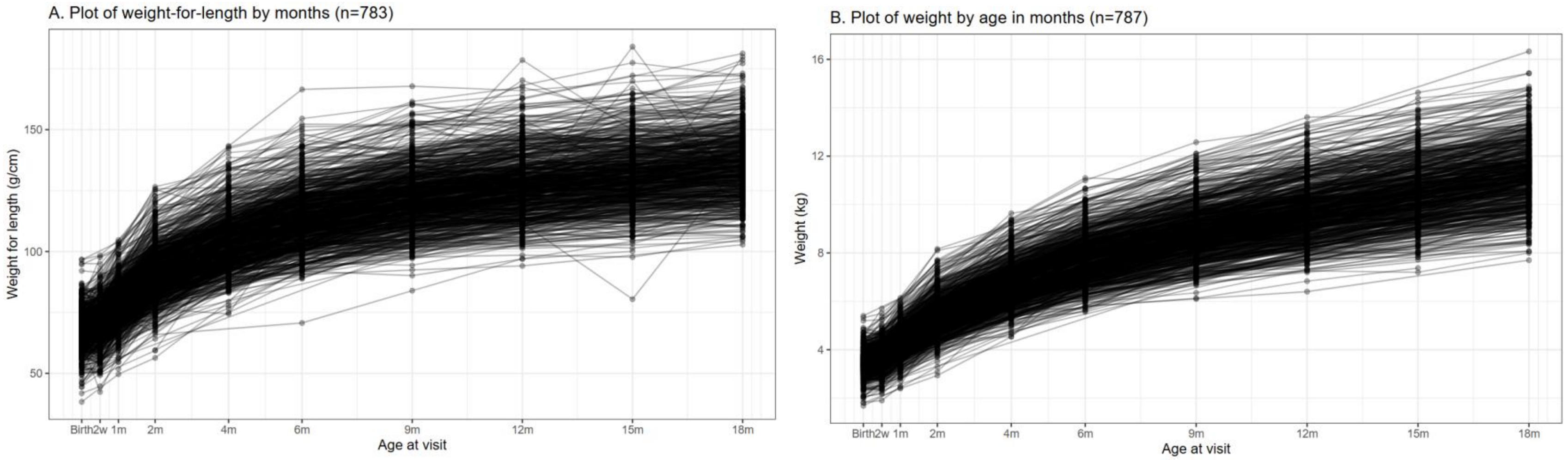

Table S1. Growth mixture model fit indices for infant weight-for-length (g/cm) in the New Hampshire Birth Cohort Study

Number of classes	Size of each class (%)	Log-likelihood	AIC	BIC	Posterior probability > 0.7 in each class (%)
Male infants (n=387)					
1	100	-11771.97	23557.93	23585.64	100
2	60.72 / 39.28	-11446.61	22919.22	22970.68	94.89 / 94.74
3	34.37 / 53.49 / 12.14	-11325.61	22689.23	22764.44	92.48 / 89.37 / 93.62
4	28.94 / 32.30 / 27.39 / 11.37	-11232.62	22515.25	22614.21	88.39 / 79.20 / 81.13 / 95.45
5	18.09 / 26.10 / 16.02 / 29.20 / 10.59	-11204.58	22471.17	22593.88	78.57 / 80.20 / 70.97 / 79.65 / 92.68
6	15.76 / 31.52 / 2.58 / 19.38 / 22.22 / 8.53	-11155.91	22385.82	22532.28	83.61 / 82.79 / 90.00 / 81.33 / 70.93 / 87.88
Female infants (n=396)					
1	100	-11497.68	23009.36	23037.23	100
2	53.79 / 46.21	-11115.87	22257.74	22309.50	92.96 / 89.62
3	36.62 / 16.67 / 46.72	-10974.36	21986.71	22062.36	90.34 / 89.39 / 91.89
4	35.35 / 7.07 / 44.95 / 12.63	-10887.53	21825.06	21924.59	91.43 / 82.14 / 87.64 / 96.00
5	33.33 / 33.08 / 3.03 / 16.92 / 13.64	-10834.15	21730.29	21853.71	88.64 / 80.15 / 91.67 / 76.12 / 92.59
6	33.08 / 2.27 / 38.89 / 5.81 / 10.35 / 9.60	-10801.21	21676.42	21823.73	90.84 / 88.89 / 83.77 / 73.91 / 80.49 / 86.84

Abbreviations: AIC, Akaike information criterion; BIC, Bayesian information criterion.

Table S2. Growth mixture model fit indices for infant weight (kg) in the New Hampshire Birth Cohort Study

Number of classes	Size of each class (%)	Log-likelihood	AIC	BIC	Posterior probability > 0.7 in each class (%)
Male infants (n=386)					
1	100	-3422.09	6858.17	6885.86	100
2	56.48 / 43.52	-2833.62	5693.25	5744.67	94.95 / 91.67
3	32.90 / 55.44 / 11.66	-2584.85	5207.71	5282.87	95.28 / 91.12 / 88.89
4	36.01 / 22.80 / 30.57 / 10.62	-2475.33	5000.67	5099.56	88.49 / 80.68 / 86.44 / 90.24
5	22.54 / 17.62 / 29.79 / 21.24 / 8.81	-2374.35	4810.71	4933.34	94.25 / 82.35 / 88.70 / 81.71 / 100.00
6	22.80 / 11.66 / 26.17 / 16.32 / 13.99 / 9.07	-2330.95	4735.91	4882.28	93.18 / 84.44 / 77.23 / 80.95 / 83.33 / 91.43
Female infants (n=401)					
1	100	-3209.28	6432.56	6460.52	100
2	53.87 / 46.13	-2509.12	5044.24	5096.16	96.30 / 95.14
3	46.63 / 33.17 / 20.20	-2271.50	4580.99	4656.88	87.17 / 93.23 / 92.59
4	36.41 / 5.99 / 42.89 / 14.71	-2173.91	4397.81	4497.66	90.41 / 87.50 / 90.12 / 93.22
5	16.21 / 37.41 / 6.23 / 30.42 / 9.73	-2036.30	4134.60	4258.42	90.77 / 90.67 / 96.00 / 86.07 / 97.44
6	17.46 / 27.68 / 11.97 / 5.49 / 28.43 / 8.98	-1958.42	3990.84	4138.62	97.14 / 78.38 / 81.25 / 95.45 / 90.35 / 100.00

Abbreviations: AIC, Akaike information criterion; BIC, Bayesian information criterion.

Table S3. Number of observations and distribution of weight-for-length (g/cm) by infant's growth mixture modeling (GMM) trajectory class

	**Stable-slow (n=112 male; n=140 female)**		**Late-moderate (n=125 male; n=28 female)**		**Stable-moderate (n=106 male; n=178 female)**		**Rapid (n=44 male; n=50 female)**	
**Time points**	**Median (IQR)**	**N**	**Median (IQR)**	**N**	**Median (IQR)**	**N**	**Median (IQR)**	**N**
Male infants (n=387)								
Birth	70.2 (67.3, 76.1)	112	67.2 (62.9, 71.9)	122	69.5 (64.6, 73.4)	106	65.5 (60.6, 70.6)	42
2 weeks	73.1 (69.1, 76.6)	79	67.8 (62.9, 73.5)	96	74.3 (68.6, 77.6)	75	66.7 (58.4, 72.4)	27
1 month	83.6 (78.1, 89.8)	49	74.2 (68.9, 79.4)	62	85.5 (80.5, 90.4)	52	69.4 (64.3, 81.0)	25
2 months	93.7 (87.5, 101)	107	88.7 (82.2, 94.1)	117	102 (96.1, 110)	100	93.2 (85.7, 104)	43
4 months	104 (95.6, 110)	109	102 (97.3, 107)	123	116 (111, 123)	100	114 (107, 122)	43
6 months	112 (114, 117)	109	112 (107, 117)	121	127 (122, 133)	101	124 (119, 136)	44
9 months	117 (111, 123)	103	122 (116, 127)	119	134 (129, 140)	94	139 (132, 152)	41
12 months	123 (115, 127)	109	129 (123, 134)	117	139 (132, 144)	99	150 (143, 158)	38
15 months	125 (119, 129)	90	134 (128, 142)	96	142 (133, 147)	86	153 (145, 160)	32
18 months	130 (123, 135)	94	139 (133, 146)	105	143 (137, 148)	87	160 (150, 163)	33
Female infants (n=396)								
Birth	69.2 (65.1, 76.4)	139	69.8 (66.2, 73.2)	28	65.3 (60.5, 69.8)	177	65.3 (60.4, 71.1)	47
2 weeks	70.9 (67.5, 76.5)	87	69.4 (66.1, 76.9)	18	67.9 (62.7, 71.5)	140	67.3 (62.1, 69.8)	34
1 month	78.9 (74.5, 82.7)	56	72.4 (69.2, 81.2)	9	75.4 (71.1, 79.9)	82	74.1 (68.8, 78.4)	22
2 months	86.6 (82.4, 93.2)	131	81.8 (78.6, 89.3)	25	88.5 (82.7, 94.8)	174	86.0 (80.8, 91.0)	47
4 months	95.6 (92.0, 104)	128	94.0 (86.6, 98.2)	27	103 (97.2, 109)	171	105 (99.1, 111)	43
6 months	104 (99.8, 110)	129	102 (99.1, 109)	28	112 (108, 119)	173	121 (116, 125)	47
9 months	110 (105, 117)	131	119 (112, 123)	27	121 (116, 126)	165	131 (126, 136)	45
12 months	115 (111, 121)	135	129 (124, 132)	28	126 (120, 130)	165	140 (132, 144)	48
15 months	120 (114, 124)	111	136 (131, 142)	26	129 (124, 134)	125	144 (136, 149)	38
18 months	122 (116, 128)	118	146 (138, 148)	26	131 (127, 137)	135	150 (140, 154)	41

Table S4. Number of observations and distribution of weight (kg) by infant's growth mixture modeling (GMM) trajectory class

	**Stable-slow (n=139 male; n=146 female)**		**Late-moderate (n=88 male; n=24 female)**		**Stable-moderate (n=118 male; n=172 female)**		**Rapid (n=41 male; n=59 female)**		**WHO growth chart**
**Time points**	**Median (IQR)**	**N**	**Median (IQR)**	**N**	**Median (IQR)**	**N**	**Median (IQR)**	**N**	**Median**
Male infants (n=386)									
Birth	3.54 (3.25, 3.80)	139	3.43 (3.19, 3.76)	88	3.61 (3.31, 3.89)	118	3.49 (3.24, 3.70)	41	3.3
2 weeks	3.74 (3.36, 4.03)	115	3.68 (3.35, 3.90)	76	3.91 (3.43, 4.19)	97	3.77 (3.52, 4.06)	31	3.8
1 month	4.42 (3.90, 4.90)	69	4.18 (3.66, 4.51)	47	4.79 (4.10, 5.12)	58	4.22 (3.72, 4.93)	23	4.5
2 months	5.50 (4.96, 5.89)	134	5.34 (4.93, 5.67)	85	6.05 (5.56, 6.62)	110	6.08 (5.22, 6.42)	40	5.6
4 months	6.62 (6.09, 7.18)	136	6.70 (6.37, 7.11)	88	7.52 (7.12, 8.09)	113	7.75 (7.24, 8.34)	38	7.0
6 months	7.40 (7.01, 7.94)	136	7.85 (7.40, 8.16)	86	8.73 (8.36, 9.22)	112	9.12 (8.62, 9.95)	41	7.9
9 months	8.42 (7.95, 8.96)	129	9.06 (8.70, 9.40)	85	9.83 (9.50, 10.3)	104	11.0 (10.2, 11.6)	40	8.9
12 months	9.30 (8.60, 9.70)	135	10.2 (9.72, 10.6)	84	10.7 (10.2, 11.2)	107	12.2 (11.5, 12.7)	37	9.6
15 months	9.91 (9.39, 10.4)	106	11.1 (10.7, 11.7)	76	11.2 (10.7, 11.9)	93	13.0 (12.3, 13.6)	30	10.3
18 months	10.6 (9.99, 11.2)	114	12.2 (11.6, 12.7)	78	11.8 (11.3, 12.4)	96	13.8 (13.3, 14.5)	33	10.9
Female infants (n=401)									
Birth	3.37 (3.09, 3.70)	146	3.61 (3.30, 3.87)	24	3.40 (3.10, 3.57)	172	3.54 (3.16, 3.76)	59	3.2
2 weeks	3.60 (3.33, 3.94)	111	3.78 (3.58, 4.03)	20	3.57 (3.25, 3.88)	151	3.60 (3.13, 3.91)	50	3.6
1 month	4.08 (3.71, 4.42)	69	4.11 (3.82, 4.61)	12	4.14 (3.76, 4.39)	83	4.26 (4.08, 4.72)	24	4.2
2 months	4.91 (4.60, 5.26)	136	4.92 (4.65, 5.37)	23	5.20 (4.79, 5.67)	170	5.24 (4.77, 5.76)	56	5.1
4 months	5.93 (5.66, 6.32)	136	6.04 (5.55, 6.29)	23	6.61 (6.20, 7.07)	162	6.76 (6.44, 7.41)	52	6.4
6 months	6.82 (6.46, 7.14)	137	6.91 (6.57, 7.41)	24	7.64 (7.30, 8.09)	167	8.21 (7.96, 8.75)	56	7.3
9 months	7.71 (7.26, 8.17)	136	8.59 (8.19, 8.81)	23	8.71 (8.35, 9.12)	162	9.64 (9.27, 10.2)	53	8.2
12 months	8.42 (8.08, 8.85)	141	9.87 (9.42, 10.1)	24	9.53 (9.15, 9.92)	162	10.8 (10.4, 11.4)	54	8.9
15 months	9.24 (8.80, 9.53)	117	11.0 (10.6, 11.3)	21	10.3 (9.90, 10.7)	125	11.9 (11.3, 12.2)	43	9.6
18 months	9.76 (9.24, 10.2)	122	12.0 (11.6, 12.5)	21	11.1 (10.6, 11.4)	135	12.7 (12.2, 13.1)	47	10.2

Table S5. Selection of a reference weight (kg) growth trajectory group using sum of squares

**Time points**	**Stable-slow**	**Late-moderate**	**Stable-moderate**	**Rapid**
	**(median weight from the NHBCS – median weight by the WHO growth chart)^2**			
Male infants (n=386)				
Birth	0.06	0.02	0.10	0.04
2 weeks	0.00	0.01	0.01	0.00
1 month	0.01	0.10	0.08	0.08
2 months	0.01	0.07	0.20	0.23
4 months	0.14	0.09	0.27	0.56
6 months	0.25	0.00	0.69	1.49
9 months	0.23	0.03	0.86	4.41
12 months	0.09	0.36	1.21	6.76
15 months	0.15	0.64	0.81	7.29
18 months	0.09	1.69	0.81	8.41
**Sum**	**1.03**	**3.01**	**5.05**	**29.27**
Female infants (n=401)				
Birth	0.03	0.17	0.04	0.12
2 weeks	0.00	0.03	0.00	0.00
1 month	0.01	0.01	0.00	0.00
2 months	0.04	0.03	0.01	0.02
4 months	0.22	0.13	0.04	0.13
6 months	0.23	0.15	0.12	0.83
9 months	0.24	0.15	0.26	2.07
12 months	0.23	0.94	0.40	3.61
15 months	0.13	1.96	0.49	5.29
18 months	0.19	3.24	0.81	6.25
**Sum**	**1.32**	**6.82**	**2.17**	**18.32**

The sum of squared differences between median weight from the NHBCS and WHO growth chart was calculated from birth to 18 months of age.

Figure S4. Weight (kg) growth trajectories for male and female infants*

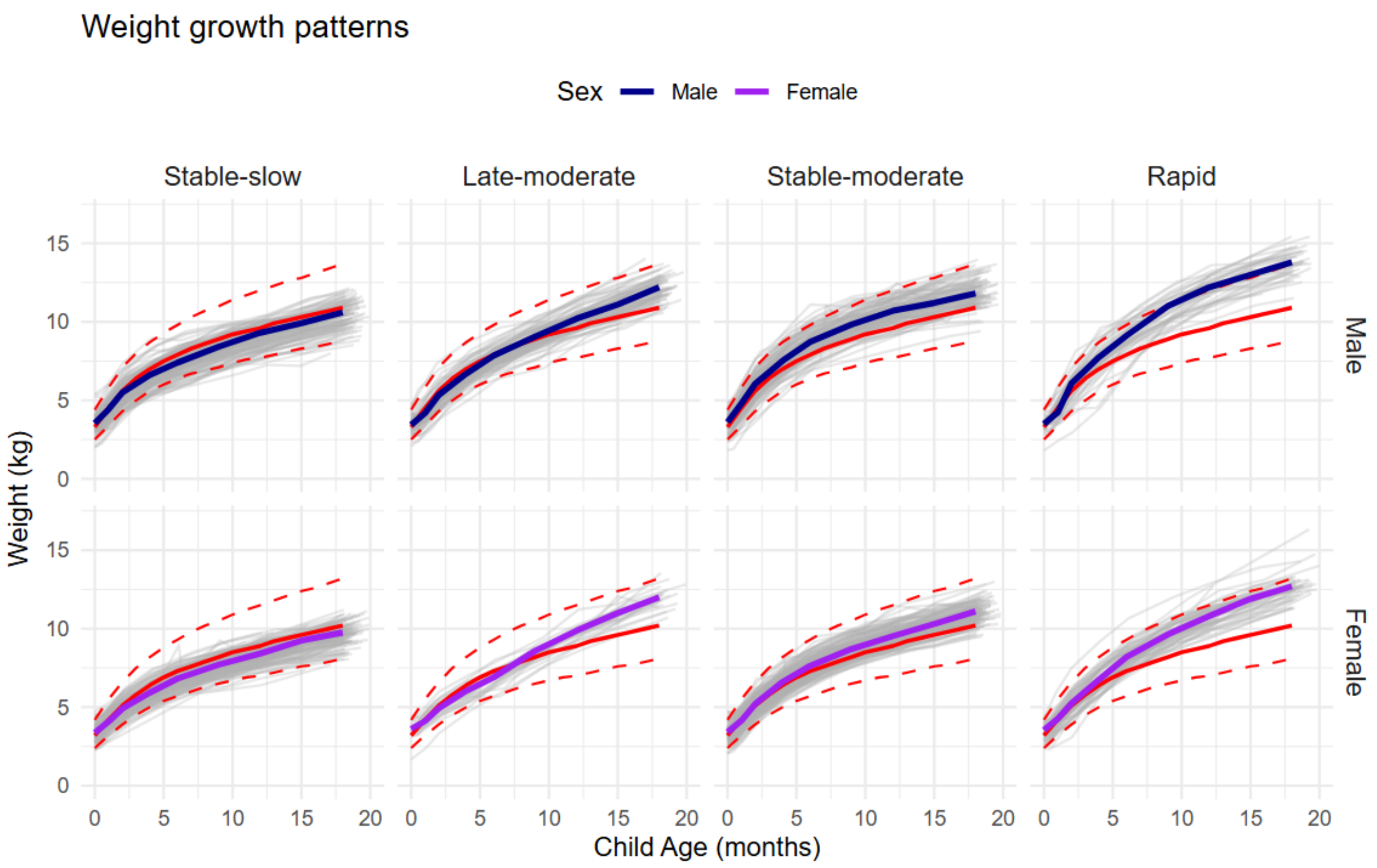

*The weight (kg) trajectory patterns estimated by sex-stratified growth mixture modeling. Dark blue or purple colors indicate median weight by age in the New Hampshire Birth Cohort Study. Grey color indicates individual growth patterns from birth to 18 months of age in the New Hampshire Birth Cohort Study. Red lines indicate the median values from the World Health Organization (WHO) weight-for-age chart. Dashed red lines indicate -2 standard deviation (SD) and +2 SD from the WHO weight-for-age charts.

Table S6. Overlap between infant growth trajectory memberships estimated using weight (kg; n=787) versus weight-for-length (g/cm; n=783)

**Merged data size: n=778**		**Weight (kg) growth trajectory membership**			
		**Stable-slow**	**Late-moderate**	**Stable-moderate**	**Rapid**
**Weight-for-length (g/cm) trajectory membership**	**Stable-slow**	217	4	29	0
	**Late-moderate**	36	93	17	5
	**Stable-moderate**	29	7	230	18
	**Rapid**	0	6	11	76

Table S7. Descriptive characteristics of mother-child pairs included (n=783) versus excluded from analysis (n=183)

**Characteristic**	**Those included (n=783)**	**Those excluded (n=183)**
Maternal age at enrollment (years)	31.6±4.5	31.4±5.1
Maternal education		
High school graduate or less	76 (9.7)	18 (9.8)
Any college graduate	462 (59.0)	95 (51.9)
Post-graduate	245 (31.3)	49 (26.8)
Missing	0 (0)	21 (11.5)
Maternal marital status		
Married	701 (89.5)	142 (77.6)
Not married	82 (10.5)	20 (10.9)
Missing	0 (0)	21 (11.5)
Maternal parity		
Primiparous	322 (41.1)	65 (35.5)
Parous	461 (58.9)	101 (55.2)
Missing	0 (0)	17 (9.3)
Maternal smoking in pregnancy		
Never smoked and never exposed to secondhand smoke	642 (82.0)	133 (72.7)
Former or current smoker, or exposed to secondhand smoke during pregnancy	141 (18.0)	33 (18.0)
Missing	0 (0)	17 (9.3)
Maternal fish/seafood consumption		
Never or less than once per month	171 (21.8)	34 (18.6)
Once to three times per month	497 (63.5)	95 (51.9)
Once per week or more	115 (14.7)	12 (6.6)
Missing	0 (0)	42 (23.0)
Maternal pre-pregnancy BMI (kg/m^2)	25.8±5.4	25.1±4.7
BMI<25 kg/m^2, N (%)	420 (53.6)	101 (55.2)
BMI≥25 kg/m^2, N (%)	363 (46.4)	74 (40.4)
Missing	0 (0)	8 (4.4)
Maternal urinary arsenobetaine level (μg/L), median (25th, 75th percentiles)	0.7 (0.1, 3.6)	0.9 (0.1, 3.6)
Missing	65 (8.3)	23 (12.6)
Infant sex		
Male	387 (49.4)	94 (51.4)

Female	396 (50.6)	89 (48.6)
Infant birth weight (g)	3451.0±517.6	3437.4±554.8
Missing	0 (0)	15 (8.2)
Infant gestational age at birth (weeks)	39.1±1.7	38.9±1.8
Sex-specific birth weight-for-gestational age z-score	0.2±1.1	0.2±1.1
Missing	3 (0.4)	19 (10.4)
Breastfeeding		
One year of breastfeeding or more	405 (51.7)	63 (34.4)
Less than one year of breastfeeding	285 (36.4)	76 (41.5)
Missing	93 (11.9)	44 (24.0)

The numbers are shown as mean (standard deviation) or N (%) for continuous or categorical variables, respectively.

Table S8. Detailed distributions of selected elements in the New Hampshire Birth Cohort Study (n=783)

**Element**	**Minimum**	**5th percentile**	**25th percentile**	**50th percentile (median)**	**75th percentile**	**95th percentile**	**Maximum**
As, µg/g	<0.01	0.02	0.04	0.05	0.08	0.21	0.74
Hg, µg/g	<0.01	0.01	0.04	0.08	0.16	0.39	1.59
Pb, µg/g	<0.01	0.02	0.06	0.11	0.22	0.69	11.46
Cu, µg/g	0.11	0.11	3.25	3.87	4.71	10.23	105.62
Mn, µg/g	<0.01	0.07	0.16	0.32	0.63	1.74	19.95
Se, µg/g	0.45	0.80	0.91	1.00	1.09	1.27	3.47

Table S9. Relative risk ratios (95% CI) for weight-for-length growth trajectory patterns by maternal toenail toxic element concentrations (continuous) in the New Hampshire Birth Cohort Study (n=783)

**Risk Ratios**	**Male infants (n=387)**	**Female infants (n=396)**
As		
Stable-slow	1.00 (reference)	1.00 (reference)
Late-moderate	1.05 (0.70, 1.58)	NC
Stable-moderate	0.87 (0.59, 1.30)	NC
Rapid	0.77 (0.49, 1.20)	NC
Hg		
Stable-slow	1.00 (reference)	1.00 (reference)
Late-moderate	1.78 (1.06, 3.00)	1.44 (0.78, 2.68)
Stable-moderate	1.64 (1.08, 2.47)	1.32 (0.77, 2.26)
Rapid	1.49 (0.99, 2.25)	1.35 (0.79, 2.30)
Pb		
Stable-slow	1.00 (reference)	1.00 (reference)
Late-moderate	1.14 (0.80, 1.62)	1.05 (0.81, 1.37)
Stable-moderate	1.05 (0.68, 1.63)	1.12 (0.79, 1.59)
Rapid	1.39 (1.00, 1.94)	0.92 (0.56, 1.51)

Relative risk ratios were estimated comparing each weight-for-length growth trajectory group to referent stable-slow growth for two-fold increase in maternal toenail concentrations. Adjusted for maternal age at enrollment, educational attainment level, marital status, tobacco exposure during pregnancy, seafood consumption during pregnancy, pre-pregnancy body mass index (kg/m^2), and parity. Abbreviations: As, arsenic; Hg, mercury; NC, model did not converge; Pb, lead.

Table S10. Relative risk ratios (95% CI) for weight-for-length growth trajectory patterns by maternal toenail toxic element concentrations (tertiles) in the New Hampshire Birth Cohort Study (n=783)

**Risk Ratios**		**Male infants (n=387)**	**Female infants (n=396)**
As			
Stable-slow		1.00 (reference)	1.00 (reference)
Late-moderate	The middle vs. low tertiles (Ref)	0.88 (0.42, 1.85)	1.22 (0.43, 3.46)
	The high vs. low tertiles (Ref)	2.00 (0.85, 4.73)	0.64 (0.20, 2.10)
Stable-moderate	The middle vs. low tertiles (Ref)	1.13 (0.49, 2.62)	1.74 (0.91, 3.32)
	The high vs. low tertiles (Ref)	2.48 (0.98, 6.24)	1.14 (0.59, 2.20)
Rapid	The middle vs. low tertiles (Ref)	1.50 (0.56, 4.04)	1.64 (0.61, 4.44)
	The high vs. low tertiles (Ref)	2.90 (0.96, 8.73)	1.70 (0.65, 4.42)
Hg			
Stable-slow		1.00 (reference)	1.00 (reference)
Late-moderate	The middle vs. low tertiles (Ref)	1.55 (0.69, 3.48)	1.57 (0.46, 5.36)
	The high vs. low tertiles (Ref)	2.72 (1.11, 6.66)	2.24 (0.62, 8.07)
Stable-moderate	The middle vs. low tertiles (Ref)	2.36 (0.85, 6.55)	2.18 (0.65, 7.34)
	The high vs. low tertiles (Ref)	5.49 (1.77, 17.00)	1.92 (0.53, 6.93)
Rapid	The middle vs. low tertiles (Ref)	2.28 (1.02, 5.07)	2.34 (0.56, 9.83)
	The high vs. low tertiles (Ref)	3.72 (1.43, 9.67)	2.31 (0.54, 9.89)
Pb			
Stable-slow		1.00 (reference)	1.00 (reference)
Late-moderate	The middle vs. low tertiles (Ref)	1.11 (0.40, 3.02)	NC
	The high vs. low tertiles (Ref)	0.88 (0.32, 2.38)	1.56 (0.54, 4.53)
Stable-moderate	The middle vs. low tertiles (Ref)	2.25 (0.94, 5.39)	NC
	The high vs. low tertiles (Ref)	1.85 (0.82, 4.17)	1.47 (0.50, 4.35)
Rapid	The middle vs. low tertiles (Ref)	2.34 (0.93, 5.87)	NC
	The high vs. low tertiles (Ref)	1.22 (0.49, 3.01)	1.43 (0.30, 6.93)

Relative risk ratios were estimated comparing each weight-for-length growth trajectory group to referent stable-slow growth for the middle or high tertile of maternal toenail concentrations compared to the low tertile. Adjusted for maternal age at enrollment, educational attainment level, marital status, tobacco exposure during pregnancy, seafood consumption during pregnancy, pre-pregnancy body mass index (kg/m^2), and parity. Abbreviations: As, arsenic; Hg, mercury; NC, model did not converge; Pb, lead.

Table S11. Relative risk ratios (95% CI) for weight-for-length growth trajectory patterns by maternal toenail essential element concentrations (tertiles) in the New Hampshire Birth Cohort Study (n=783)

**Risk Ratios**		**Male infants (n=387)**	**Female infants (n=396)**
Cu			
Stable-slow		1.00 (reference)	1.00 (reference)
Late-moderate	The low vs. middle tertiles (Ref)	0.57 (0.24, 1.35)	0.34 (0.11, 1.07)
	The high vs. middle tertiles (Ref)	0.47 (0.20, 1.13)	0.57 (0.19, 1.69)
Stable-moderate	The low vs. middle tertiles (Ref)	0.73 (0.33, 1.60)	0.59 (0.30, 1.15)
	The high vs. middle tertiles (Ref)	0.46 (0.20, 1.06)	1.10 (0.58, 2.09)
Rapid	The low vs. middle tertiles (Ref)	0.26 (0.09, 0.74)	0.65 (0.27, 1.54)
	The high vs. middle tertiles (Ref)	0.25 (0.09, 0.73)	0.61 (0.25, 1.50)
Mn			
Stable-slow		1.00 (reference)	1.00 (reference)
Late-moderate	The low vs. middle tertiles (Ref)	0.97 (0.37, 2.57)	NC
	The high vs. middle tertiles (Ref)	0.64 (0.26, 1.57)	NC
Stable-moderate	The low vs. middle tertiles (Ref)	2.43 (1.11, 5.35)	NC
	The high vs. middle tertiles (Ref)	0.83 (0.36, 1.92)	NC
Rapid	The low vs. middle tertiles (Ref)	1.23 (0.57, 2.65)	NC
	The high vs. middle tertiles (Ref)	0.63 (0.29, 1.33)	NC
Se			
Stable-slow		1.00 (reference)	1.00 (reference)
Late-moderate	The low vs. middle tertiles (Ref)	NC	1.58 (0.35, 7.05)
	The high vs. middle tertiles (Ref)	NC	0.54 (0.18, 1.57)
Stable-moderate	The low vs. middle tertiles (Ref)	NC	0.86 (0.18, 4.02)
	The high vs. middle tertiles (Ref)	NC	0.47 (0.16, 1.35)
Rapid	The low vs. middle tertiles (Ref)	NC	2.42 (0.45, 13.01)
	The high vs. middle tertiles (Ref)	NC	1.46 (0.38, 5.55)

Relative risk ratios were estimated comparing each weight-for-length growth trajectory group to referent stable-slow growth for the low or high tertile of maternal toenail concentrations compared to the middle tertile. Adjusted for maternal age at enrollment, educational attainment level, marital status, tobacco exposure during pregnancy, seafood consumption during pregnancy, pre-pregnancy body mass index (kg/m^2), and parity. Abbreviations: Cu, copper; Mn, manganese; NC, model did not converge; Se, selenium.

Table S12. Relative risk ratios (95% CI) for weight-for-length growth trajectory patterns by maternal toenail toxic metal (continuous) and essential metal concentrations (tertiles) in the New Hampshire Birth Cohort Study, using a two-stage approach (n=783)

		Male infants (n=387)		Female infants (n=396)	
		**Unadjusted**	**Adjusted[a]**	**Unadjusted**	**Adjusted[a]**
As					
Stable-slow		1.00 (reference)	1.00 (reference)	1.00 (reference)	1.00 (reference)
Late-moderate		1.08 (0.84, 1.40)	1.11 (0.84, 1.46)	0.90 (0.60, 1.35)	0.97 (0.64, 1.49)
Stable-moderate		1.01 (0.77, 1.32)	0.99 (0.74, 1.31)	0.89 (0.71, 1.11)	0.91 (0.72, 1.14)
Rapid		1.12 (0.79, 1.58)	1.12 (0.78, 1.61)	0.97 (0.70, 1.34)	0.99 (0.71, 1.40)
Hg					
Stable-slow		1.00 (reference)	1.00 (reference)	1.00 (reference)	1.00 (reference)
Late-moderate		1.16 (0.89, 1.52)	1.39 (0.99, 1.94)	0.98 (0.67, 1.43)	0.74 (0.46, 1.21)
Stable-moderate		1.30 (0.98, 1.73)	1.41 (1.00, 2.01)	1.06 (0.86, 1.31)	1.00 (0.77, 1.31)
Rapid		1.51 (1.03, 2.23)	1.81 (1.12, 2.94)	1.23 (0.89, 1.70)	1.14 (0.76, 1.69)
Pb					
Stable-slow		1.00 (reference)	1.00 (reference)	1.00 (reference)	1.00 (reference)
Late-moderate		0.82 (0.64, 1.07)	0.79 (0.60, 1.03)	0.80 (0.52, 1.23)	0.87 (0.55, 1.35)
Stable-moderate		0.78 (0.60, 1.02)	0.77 (0.58, 1.02)	0.98 (0.78, 1.23)	0.99 (0.78, 1.25)
Rapid		0.83 (0.59, 1.18)	0.82 (0.57, 1.18)	1.17 (0.85, 1.61)	1.14 (0.82, 1.59)
Cu					
Stable-slow		1.00 (reference)	1.00 (reference)	1.00 (reference)	1.00 (reference)
Late-moderate	The low vs. middle tertiles (Ref)	1.00 (0.55, 1.83)	0.98 (0.53, 1.83)	0.66 (0.24, 1.85)	0.61 (0.21, 1.77)
	The high vs. middle tertiles (Ref)	0.90 (0.47, 1.70)	0.91 (0.47, 1.75)	0.55 (0.21, 1.41)	0.53 (0.20, 1.41)
Stable-moderate	The low vs. middle tertiles (Ref)	1.39 (0.72, 2.69)	1.42 (0.73, 2.80)	1.31 (0.75, 2.31)	1.39 (0.78, 2.48)
	The high vs. middle tertiles (Ref)	1.73 (0.89, 3.38)	1.86 (0.94, 3.69)	0.74 (0.43, 1.27)	0.76 (0.44, 1.33)
Rapid	The low vs. middle tertiles (Ref)	0.56 (0.24, 1.28)	0.50 (0.21, 1.19)	0.94 (0.42, 2.12)	1.05 (0.46, 2.41)
	The high vs. middle tertiles (Ref)	0.55 (0.23, 1.33)	0.55 (0.22, 1.37)	0.69 (0.32, 1.49)	0.69 (0.31, 1.52)
Mn					
Stable-slow		1.00 (reference)	1.00 (reference)	1.00 (reference)	1.00 (reference)
Late-moderate	The low vs. middle tertiles (Ref)	1.12 (0.60, 2.11)	1.18 (0.61, 2.25)	1.42 (0.50, 4.00)	1.26 (0.44, 3.60)
	The high vs. middle tertiles (Ref)	0.77 (0.42, 1.41)	0.69 (0.36, 1.30)	1.62 (0.60, 4.37)	1.69 (0.62, 4.66)
Stable-moderate	The low vs. middle tertiles (Ref)	1.93 (1.00, 3.73)	2.06 (1.05, 4.05)	1.33 (0.77, 2.27)	1.43 (0.82, 2.49)
	The high vs. middle tertiles (Ref)	0.93 (0.48, 1.81)	0.94 (0.47, 1.87)	1.08 (0.63, 1.85)	1.19 (0.68, 2.08)
Rapid	The low vs. middle tertiles (Ref)	0.91 (0.39, 2.14)	1.03 (0.42, 2.50)	1.61 (0.66, 3.90)	1.71 (0.69, 4.26)
	The high vs. middle tertiles (Ref)	0.67 (0.29, 1.55)	0.69 (0.29, 1.65)	2.68 (1.19, 6.03)	2.97 (1.29, 6.87)
Se					
Stable-slow		1.00 (reference)	1.00 (reference)	1.00 (reference)	1.00 (reference)
Late-moderate	The low vs. middle tertiles (Ref)	0.65 (0.35, 1.22)	0.66 (0.35, 1.27)	0.85 (0.27, 2.64)	0.88 (0.27, 2.80)
	The high vs. middle tertiles (Ref)	0.74 (0.40, 1.38)	0.74 (0.39, 1.42)	2.03 (0.78, 5.29)	2.29 (0.83, 6.31)
Stable-moderate	The low vs. middle tertiles (Ref)	1.27 (0.66, 2.43)	1.27 (0.65, 2.47)	0.76 (0.44, 1.30)	0.70 (0.40, 1.23)

	The high vs. middle tertiles (Ref)	0.85 (0.43, 1.68)	0.90 (0.45, 1.82)	0.93 (0.55, 1.59)	0.95 (0.54, 1.65)
Rapid	The low vs. middle tertiles (Ref)	1.44 (0.62, 3.36)	1.41 (0.59, 3.38)	1.51 (0.65, 3.53)	1.38 (0.58, 3.29)
	The high vs. middle tertiles (Ref)	0.78 (0.30, 1.99)	0.91 (0.34, 2.38)	2.12 (0.94, 4.78)	2.13 (0.92, 4.93)

Relative risk ratios were estimated comparing each weight-for-length growth trajectory group to referent stable-slow growth for two-fold increase in maternal toenail concentrations (toxic elements) or for the low or high tertile of maternal toenail concentrations compared to the middle tertile (essential elements).
[a]Adjusted for maternal age at enrollment, educational attainment level, marital status, tobacco exposure during pregnancy, seafood consumption during pregnancy, pre-pregnancy body mass index (kg/m^2), and parity. Abbreviations: As, arsenic; Cu, copper; Hg, mercury; Mn, manganese; NC, model did not converge; Pb, lead; Se, selenium.

Table S13. Growth mixture model fit indices for infant weight-for-length (g/cm) in the New Hampshire Birth Cohort Study, with 3 knots[a]

**Number of classes**	**Size of each class (%)**	**Log-likelihood**	**AIC**	**BIC**	**Posterior probability > 0.7 in each class (%)**
Male infants (n=387)					
1	100	-12133.56	24283.13	24314.80	100
2	65.37 / 34.63	-11924.73	23879.46	23938.84	91.70 / 84.33
3	51.68 / 36.95 / 11.37	-11855.94	23755.87	23842.96	86.50 / 85.31 / 81.82
4	32.04 / 13.18 / 42.89 / 11.89	-11791.67	23641.34	23756.13	78.23 / 70.59 / 87.95 / 82.61
5	27.39 / 2.58 / 29.72 / 29.46 / 10.85	-11755.02	23582.04	23724.54	83.96 / 100.00 / 54.78 / 71.05 / 88.10
6	25.84 / 2.58 / 24.29 / 34.88 / 3.36 / 9.04	-11727.80	23541.60	23711.81	80.00 / 100.00 / 58.51 / 74.81 / 100.00 / 85.71
Female infants (n=396)					
1	100	-11824.37	23664.75	23696.6	100
2	61.36 / 38.64	-11552.62	23135.25	23194.97	91.77 / 90.85
3	34.85 / 48.48 / 16.67	-11461.20	22966.39	23053.98	90.58 / 85.94 / 80.30
4	31.57 / 3.03 / 48.48 / 16.92	-11387.05	22832.10	22947.56	85.60 / 83.33 / 84.90 / 88.06
5	29.55 / 1.52 / 46.46 / 7.58 / 14.90	-11354.12	22780.25	22923.58	76.92 / 100.00 / 80.98 / 83.33 / 81.36-
6	30.05 / 0.76 / 46.72 / 3.28 / 17.93 / 1.26	-11286.86	22659.72	22830.92	86.55 / 100.00 / 81.62 / 76.92 / 85.92 / 100.00

[a]At 1, 4.5, and 12 months of age

Table S14. Overlap between infant growth trajectory memberships estimated using weight-for-length (g/cm), comparing 2 knots and 3 knots (n=783)

		**3 knots**			
		**Stable-slow**	**Late-moderate**	**Stable-moderate**	**Rapid**
**2 knots**	**Stable-slow**	224	7	21	0
	**Late-moderate**	23	54	65	11
	**Stable-moderate**	2	0	271	11
	**Rapid**	0	2	1	91

Table S15. Difference (95% confidence interval) in weight-for-length z-score at 18 months of age by maternal toenail toxic element concentrations (continuous) in the New Hampshire Birth Cohort Study (n=783)

	**Male infants (n=387)**	**Female infants (n=396)**
As	-0.02 (-0.14, 0.10)	0.05 (-0.08, 0.17)
Hg	0.19 (0.04, 0.33)	0.04 (-0.10, 0.18)
Pb	-0.08 (-0.19, 0.04)	0.04 (-0.09, 0.18)

Adjusted for maternal age at enrollment, educational attainment level, marital status, tobacco exposure during pregnancy, seafood consumption during pregnancy, pre-pregnancy body mass index (kg/m^2), and parity. Abbreviations: As, arsenic; Hg, mercury; Pb, lead.

Table S16. Difference (95% confidence interval) in weight-for-length z-score at 18 months of age by maternal toenail essential element concentrations (tertiles) in the New Hampshire Birth Cohort Study (n=783)

		**Male infants (n=387)**	**Female infants (n=396)**
Cu	The low vs. middle tertiles (Ref)	-0.20 (-0.48, 0.07)	0.04 (-0.26, 0.34)
	The high vs. middle tertiles (Ref)	0.01 (-0.29, 0.30)	-0.03 (-0.32, 0.27)
Mn	The low vs. middle tertiles (Ref)	-0.11 (-0.39, 0.17)	0.38 (0.09, 0.68)
	The high vs. middle tertiles (Ref)	-0.18 (-0.48, 0.11)	0.22 (-0.07, 0.52)
Se	The low vs. middle tertiles (Ref)	-0.07 (-0.35, 0.21)	0.11 (-0.20, 0.42)
	The high vs. middle tertiles (Ref)	0.01 (-0.28, 0.31)	0.26 (-0.03, 0.55)

Adjusted for maternal age at enrollment, educational attainment level, marital status, tobacco exposure during pregnancy, seafood consumption during pregnancy, pre-pregnancy body mass index (kg/m^2), and parity. Abbreviations: Cu, copper; Mn, manganese; Se, selenium.

Table S17. Descriptive characteristics of mother-child pairs among those assigned to the Late-moderate growth, stratified by sex (n=153)

**Characteristic**	**Female (n=28)**	**Male (n=125)**	**P**
Maternal age at enrollment (years)	33.13±4.54	31.09±4.91	0.04
Maternal education			0.95
High school graduate or less	3 (10.7)	15 (12.0)	
Any college graduate	17 (60.7)	70 (56.0)	
Post-graduate	8 (28.6)	40 (32.0)	
Maternal marital status			0.03
Married	28 (100)	105 (84.0)	
Not married	0 (0)	20 (16.0)	
Maternal parity			0.20
Primiparous	8 (28.6)	55 (44.0)	
Parous	20 (71.4)	70 (56.0)	
Maternal smoking in pregnancy			0.08
Never smoked and never exposed to secondhand smoke	26 (92.9)	95 (76.0)	
Former or current smoker, or exposed to secondhand smoke during pregnancy	2 (7.1)	30 (24.0)	
Maternal fish/seafood consumption			0.55
Never or less than once per month	5 (17.9)	26 (20.8)	
Once to three times per month	18 (64.3)	86 (68.8)	
Once per week or more	5 (17.9)	13 (10.4)	
Maternal pre-pregnancy BMI (kg/m^2)	26.30±4.08	25.62±5.50	0.17
BMI<25 kg/m^2, N (%)	8 (28.6)	68 (54.4)	0.02
BMI≥25 kg/m^2, N (%)	20 (71.4)	57 (45.6)	
Maternal urinary arsenobetaine level (μg/L), median (25th, 75th percentiles)	1.31 (0.26, 4.98)	0.66 (0.11, 3.66)	0.31
Missing	1 (3.6)	11 (8.8)	
Infant birth weight (g)	3574.91±424.31	3409.90±528.92	0.16
Infant gestational age at birth (weeks)	39.13±1.28	38.86±1.87	0.70
Sex-specific birth weight-for-gestational age z-score	0.63±1.05	0.07±1.08	0.01
Weight-for-height z-score at 18 months	1.25±1.76	0.41±0.85	<0.01
Missing	2 (7.1)	20 (16.0)	
Breastfeeding			0.73
One year of breastfeeding or more	7 (25.0)	36 (28.8)	

Less than one year of breastfeeding	18 (64.3)	69 (55.2)	
Missing	3 (10.7)	20 (16.0)	

The numbers are shown as mean (standard deviation) or N (%) for continuous or categorical variables, respectively. P value estimated by Mann-Whitney U test for continuous variables and chi-square of Fisher's exact test for categorical variables.

Figure S5. Pearson correlations between toenail element pairs (n=783)

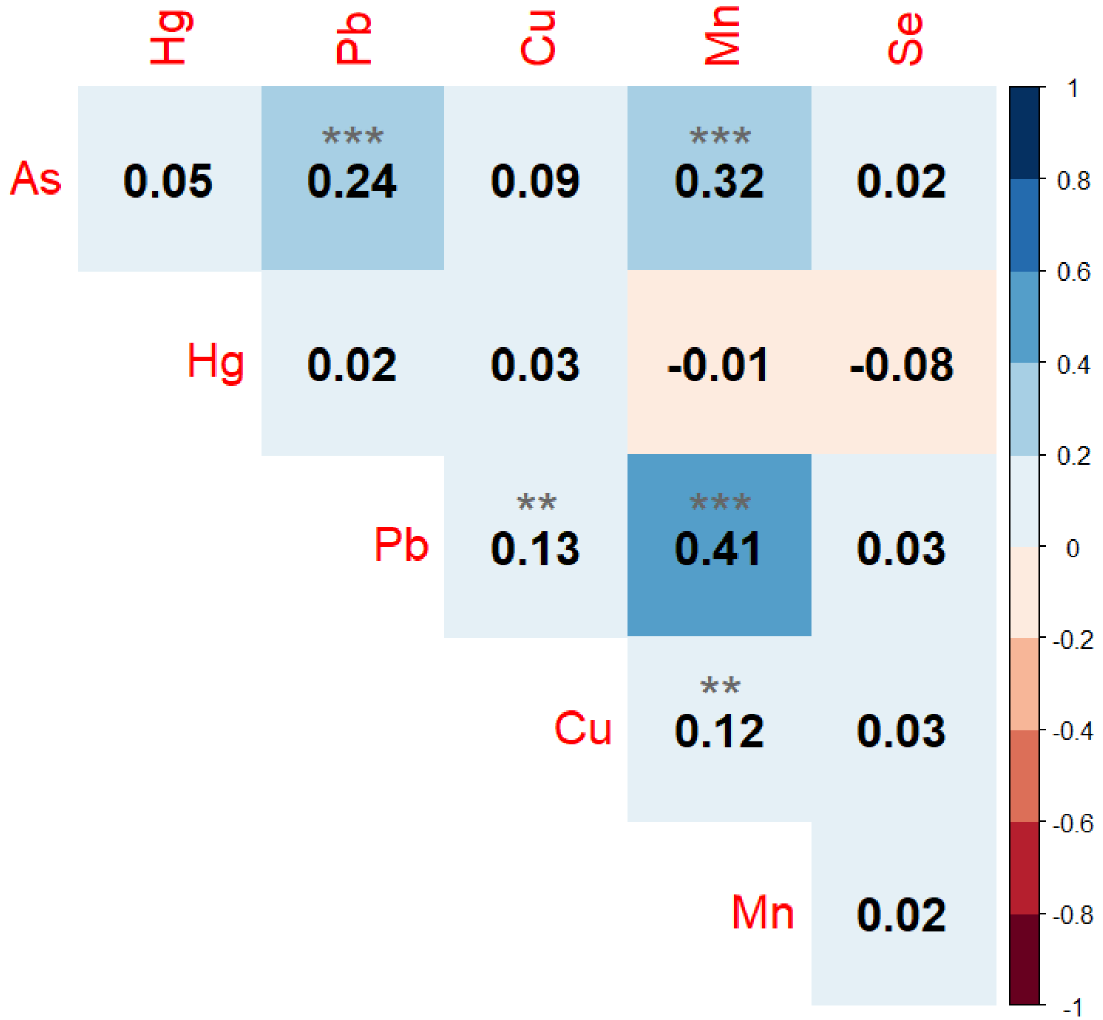

All element concentrations were log2-transformed. Positive correlations are indicated in blue shades, whereas negative correlations are indicated in red shades as shown in the key. * indicate a p-value < 0.05, ** indicates a p-value < 0.01, and *** indicates a p-value < 0.001. Abbreviations: As, arsenic; Cu, copper; Hg, mercury; Mn, manganese; Pb, lead; Se, selenium.
